# Computation-through-Dynamics Benchmark: Simulated datasets and quality metrics for dynamical models of neural activity

**DOI:** 10.1101/2025.02.07.637062

**Published:** 2025-02-20

**Authors:** Christopher Versteeg, Jonathan D. McCart, Mitchell Ostrow, David M. Zoltowski, Clayton B. Washington, Laura Driscoll, Olivier Codol, Jonathan A. Michaels, Scott W. Linderman, David Sussillo, Chethan Pandarinath

**Affiliations:** 1 Wallace H. Coulter Department of Biomedical Engineering, Emory University and Georgia Institute of Technology, Atlanta, GA, USA; 2 Center for Machine Learning, Georgia Institute of Technology, Atlanta, GA, USA; 3 Department of Brain and Cognitive Sciences, MIT, Cambridge, MA, USA; 4 Wu Tsai Neurosciences Institute, Stanford, CA, USA; 5 Department of Statistics, Stanford University, Stanford, CA, USA; 6 Allen Institute for Neural Dynamics, Seattle, WA, USA; 7 Department of Neurobiology & Biophysics, University of Washington, Seattle, WA, USA; 8 Département de Neurosciences, Faculté de Médecine, Université de Montréal, Montréal, Canada; 9 MILA, Quebec Artificial Intelligence Institute, Montréal, Canada; 10 School of Kinesiology and Health Science, Faculty of Health, York University, Toronto, ON, Canada; 11 Department of Electrical Engineering, Stanford University, Stanford, CA, USA; 12 Department of Neurosurgery, Emory University, Atlanta, GA, USA

## Abstract

A primary goal of systems neuroscience is to discover how ensembles of neurons transform inputs into goal-directed behavior, a process known as neural computation. A powerful framework for understanding neural computation uses neural dynamics – the rules that describe the temporal evolution of neural activity – to explain how goal-directed input-output transformations occur. As dynamical rules are not directly observable, we need computational models that can infer neural dynamics from recorded neural activity. We typically validate such models using synthetic datasets with known ground-truth dynamics, but unfortunately existing synthetic datasets don’t reflect fundamental features of neural computation and are thus poor proxies for neural systems. Further, the field lacks validated metrics for quantifying the accuracy of the dynamics inferred by models. The Computation-through-Dynamics Benchmark (CtDB) fills these critical gaps by providing: 1) synthetic datasets that reflect computational properties of biological neural circuits, 2) interpretable metrics for quantifying model performance, and 3) a standardized pipeline for training and evaluating models with or without known external inputs. In this manuscript, we demonstrate how CtDB can help guide the development, tuning, and troubleshooting of neural dynamics models. In summary, CtDB provides a critical platform for model developers to better understand and characterize neural computation through the lens of dynamics.

## 1 What is the computation-through-dynamics benchmark?

Discovering how the brain performs computation is the central aim of systems neuroscience. Although modern neural interfaces can now monitor hundreds or thousands of neurons simultaneously [11], we still struggle to translate these massive new datasets into interpretable accounts of neural computation. We need a language that can describe how neural populations transform inputs into goal-directed behavior. Neural dynamics - the principles governing how neural circuit activity changes over time - have gained renewed attention through advances in artificial neural network research [1, 3, 5, 15]. These principles offer a framework for connecting neural observations with neural computation [34].

A dynamical understanding of neural computation requires new methods that can estimate the (not directly observable) dynamics of neural circuits. Recent years have seen a surge in “data-driven” models that attempt to infer these dynamics by learning to reconstruct observed neural activity as the product of a model dynamical system [16, 20, 19, 25, 40, 35, 41, 55]. Unfortunately, we lack a consensus on the synthetic systems and performance criteria that are appropriate for evaluating the performance of these models. A standard set of datasets and metrics for model evaluation would facilitate model comparisons and help promising innovations be disseminated more quickly through the field.

In this manuscript, we introduce the Computation-through-Dynamics Benchmark (CtDB), a model development platform designed to help researchers assess the strengths and weaknesses of different data-driven (DD) dynamics models. As part of CtDB, we present 1) a library of new synthetic datasets that reflect goal-directed dynamical computations, 2) performance criteria and metrics sensitive to specific model failures, and 3) a public codebase for researchers to submit their models, generate new synthetic datasets, and quickly iterate during model development. We designed CtDB with a focus on modularity and extensibility so that CtDB can grow with community contributions of new datasets, models, and metrics.

This manuscript is divided into three sections: Section 2 lays out a theoretical foundation for synthetic systems and performance criteria for DD model validation. Section 3 describes the specific datasets and metrics included in CtDB. Section 4 provides illustrative examples of how CtDB datasets and metrics can help guide DD model development.

## 2 Computation-through-Dynamics: Definitions, Approach, and Challenges

### 2.1 Problem Definition

**Figure 1:**
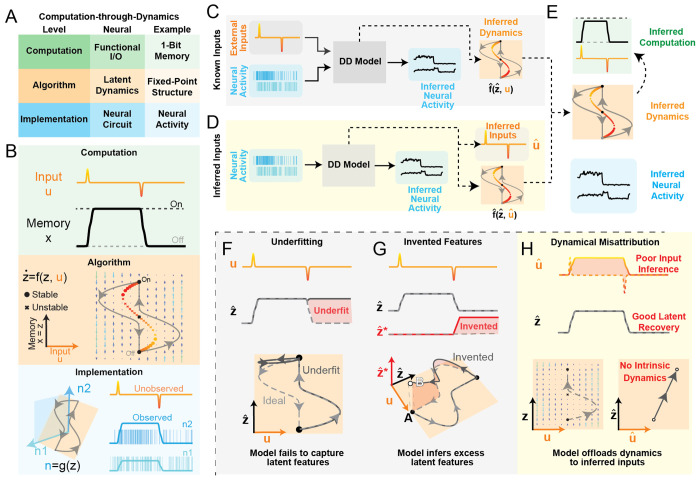
Computation-through-Dynamics framework and data-driven model failure modes **A)** Overview of Computation-through-Dynamics framework with levels, corresponding concepts in neuroscience, and specific examples from 1-Bit Memory (1BFF) task. **B)** Schematic of conceptual hierarchy of 1-Bit Memory task. Top row (green): Computation – Example of input pulses u and desired output p for a single trial of 1BFF task. Middle row (orange): Algorithm – State-space diagram of dynamical system that performs 1BFF. x-axis: external input **u**. y-axis: latent state **z**. Flow field (written as **f**) controls how **z** evolves as a function of both **z** and **u**. circles: stable fixed points, : unstable fixed points. Output **x** is generated by projection **h** acting on the latent **z**. In this example **h** is the identity function. Bottom row (blue): Implementation of latent dynamics in neural circuit. Left: Schematic of simple linear embedding of 1D latent dynamics into 2D neural activity. Inputs **u** are orthogonal to the linear embedding, implying that inputs are not directly visible in neural activity. Right: Simulated firing rates **n** and spiking activity **y** for two neurons in blue. **C)**Schematic of data-training pipeline (with known external inputs). Solid lines represent inputs and outputs from the DD model, dashed black lines represent what we hope our DD models infer indirectly. Neural observations (blue) and external inputs (orange) are provided to the dynamics model, which is optimized to reconstruct neural activity. **D)** Data-training pipeline (with inferred external inputs): Same as C, except the DD model does not receive external inputs and is instead tasked with inferring them. **E)** Our goal is to infer the computation performed by a neural circuit, but we only have access to observed neural activity. We hope that the dynamics $\hat{f}$ inferred by models in panels C,D match the true dynamics **f**, and will provide the basis for us to infer the computation performed by the circuit (green). **F-G)** Schematics of failure modes for models fit to 1BFF with supplied external inputs. **F)** Underfitting – Top row: True inputs **u** (orange/red) and true/inferred latent activity **z**/ $\hat{z}$ (solid/dashed, respectively). Red shaded area shows the activity that the underfit model fails to capture. Bottom row: State-space diagram of an ideal DD model (gray dashed line) and underfit DD model (black solid line). **G)** Invented Features – Top row: Same as F, except DD model has extra latent dimension ${\hat{z}}^{*}$, highlighted by a red shaded area. Bottom row: 3D state-space diagram of ideal DD model (gray dashed line) and DD model with invented features (solid black line). Ideal model and invented features model share fixed points (filled black circles, “A”), but the invented features model has an additional “invented” fixed point (white circle, “B”). **H)** Schematic of the dynamical misattribution failure mode, which affects models that infer external inputs. Top row: Model-inferred inputs do not match the true inputs (highlighted by red shaded area), but accurately capture the latent activity without inventing features. Bottom row: State-space diagram with flow field for ideal DD model (left) and DD model with poor input accuracy (right). In this schematic, we only show the rising bit-flip for visual clarity. With inferred inputs, DD models are not obligated to learn any intrinsic dynamics.

We first provide a brief summary of the major challenges faced by the data-driven dynamics modeling community. Any satisfying account of neural computation needs to span three conceptual levels [9]: computational, algorithmic, and implementation (Fig. 1A). In this section, we provide a high-level description of each level and illustrative examples from a simple computational system, the 1-bit flip-flop.

#### What do we mean by “neural computation”?

The neural computation level is concerned with the goal a system is trying to accomplish. Because brains evolved to generate adaptive behavior, it is impossible to fully understand a neural circuit without reference to a computational goal. We define a neural computation as a mapping from inputs **u** to outputs **x** tuned to accomplish a specific behaviorally-relevant goal (e.g., memory, sensory integration, control).

For example, we define the 1-bit flip-flop (1BFF) computation (Fig. 1B, Computation) with the following input/output mapping: the output should reflect the sign of the most recent input pulse. Importantly, a computation defines *what* is done, but not *how* it is done.

#### How are algorithms built by neural dynamics?

The second level of the hierarchy is the algorithmic level. An algorithm is a set of rules that, when followed, enact a particular computation. In the CtD framework, these algorithmic rules are built from the neural dynamics of a circuit. Formally, neuronal circuits learn a D-dimensional latent dynamical system **ż** = **f** (**z, u**) and corresponding output projection **x** = **h**(**z**) whose time-evolution approximates the input/output mapping **u** ↦ **x**.

For the 1BFF computation, we show that a relatively simple dynamical system **f** with output projection **h** (in this case, the identity function) can accomplish the desired mapping via an input-dependent flow field (Fig. 1B, Algorithm). Sufficiently large input pulses carry the state over a saddle point, which mediates the transition to a new attractor state and thereby flips the bit.

#### How are these dynamics implemented by a neural circuit?

In the brain, neural dynamics emerge as a result of the physical biology of a neural circuit (i.e., the synapses, neuromodulators, neuron types, etc.). However, we may not need to model neural dynamics using a system with the same dimensionality as the full neural space, as the dimensionality of biological neural activity is typically far lower than the total number of neurons. This suggests that relatively low-dimensional dynamics may instead be embedded into a N-dimensional neural activity space (where *N >> D*) [14, 18]. We refer to this mapping from latent space to neural activity as the embedding function **g**(**z**) [37, 18].

We simulated the embedding function *g* of the 1-bit memory system using a simple linear-exponential transformation of the one-dimensional latent state into a two-dimensional neural rates **n**, followed by sampling from a Poisson noise process to generate neural spiking activity **y** (Fig. 1B, Implementation). Importantly, external inputs **u** are not usually observable; they must be inferred from their effects on observable neural activity **y**.

### 2.2 Climbing levels: From neurons to dynamics to computation

#### Data-driven modeling:

Though an interpretable account of a neural circuit requires an understanding that spans all three conceptual levels, we typically only have direct access to observations from level of implementation – i.e., recorded neural activity **y**. Therefore, we need methods that can accurately infer algorithmic features – dynamics **f**, embedding **g**, latent activity **z** from these neural observations (Fig. 1C, D, with supplied/inferred external inputs respectively). We use $\hat{\cdot }$ to indicate such model-inferred features: for example, we refer to ground-truth dynamics as **f** and inferred dynamics as $\hat{f}$. Models that can be trusted to infer dynamics accurately (i.e., $\hat{f}≃f$) could allow us to climb the hierarchy from implementation (neural activity) to algorithm (neural dynamics), and then, hopefully, from algorithm to computation (Fig. 1E).

In recent years, a new class of “data-driven” (DD) models has emerged that are trained to reconstruct recorded neural activity as a product of low-dimensional dynamics and embedding models ($\hat{f}$, $\hat{g}$, respectively) [16, 20, 19, 25, 40, 39, 49, 47, 50, 35, 41, 48, 29, 36]. DD models are typically rated by their ability to reconstruct simulated neural activity from synthetic systems with known ground-truth dynamics **f**. Unfortunately, neural reconstruction can be an unreliable indicator of how accurately the model has captured the underlying system, and commonly-used synthetic systems lack many features that are fundamental to neural circuits [51, 55, 39]. Lastly, our lack of a common framework of synthetic systems and performance criteria for model validation makes it difficult to compare previously published models or test new architectures.

The primary goal of CtDB is to help the field converge to a theoretically-grounded collection of synthetic systems and performance criteria that will accelerate our progress towards DD models that can accurately and reliably infer neural dynamics from recorded neural activity.

#### Synthetic systems as proxies for neural circuits:

Following the lead of the broader dynamics modeling community, most neural dynamics models have been validated using synthetic systems drawn from a class of well-characterized, low-dimensional chaotic attractors such as Lorenz or Arneodo [2]. At first glance, these chaotic attractors seem well-suited for dynamical model validation. First, they are well-understood and identifiable, having no external inputs. Second, they exhibit chaotic dynamics – i.e., small changes in the system state can have a large impact on how the system develops over time – and therefore present a formidable modeling challenge. Finally, they are low-dimensional, which makes model training less computationally expensive and the results more easily interpretable.

Unfortunately, the features that make these systems appealing test-cases for generic dynamics models make them poor proxies for neural circuits that perform computation. First, chaotic attractors don’t “do” anything, lacking both the intended computation and external inputs that are fundamental features of goal-oriented neural circuits. Second, though some evidence exists that in some contexts chaos may have beneficial qualities – e.g., during learning, amplifying small signals in sensory integration tasks [13, 10, 59], chaos in dynamical systems trained to perform tasks is typically lower than these chaotic attractors [13, 8], presumably because unpredictability is anathema to behavioral stability. Finally, while the dimensionality of neural dynamics is still an open question, our highly expressive behavioral repertoire suggests that neural dynamics are likely much higher-dimensional than these low-D attractors. Therefore, we need the systems used to validate our models to be *computational* (reflecting a goal-directed input-output transformation), *regular* (not overly chaotic), and *dimensionally-rich*.

In Section 3.3, we describe how we obtain proxy systems with these properties by training dynamics models to perform specific tasks; we call these models “task-trained” (TT) models to distinguish them from the “data-driven” (DD) models that are trained to reconstruct neural activity.

#### Performance criteria:

DD models are usually trained to reconstruct recorded neural activity, but recent work has shown that even near-perfect reconstruction does not imply that inferred dynamics are an accurate estimate of the underlying system (i.e., $\hat{n}≃n$ does not imply $\hat{f}≃f$) [46, 57]. To address this problem, we have identified three key performance criteria that can collectively provide a more holistic assessment of DD model performance: *reconstruction, simplicity*, and *input accuracy*.

In this section, we provide intuition for the role of these criteria in model selection, while in Section 3.4, we provide formal definitions of specific metrics for quantifying model performance on each criterion.

##### Reconstruction:

The first and simplest performance criterion is reconstruction, or the extent to which a model can re-generate neural activity from trials held-out from the training set. Poor reconstruction can be a symptom of an important mode of model failure called underfitting. Underfitting occurs when the model fails to capture the latent features **z** that underlie the observed neural activity patterns **y**, often because the model lacks computational capacity, is over-regularized, or has had insufficient time to train. In the last few years, reconstruction has improved dramatically as the field has moved from simple linear dynamics to complex, stacked state-space models [19, 53, 45].

In Figure 1F, we show a hypothetical DD model that accurately captures the latent activity associated with the positive bit-flip (0 → 1), but fails to capture the latent activity associated with the negative bit-flip (1 → 0). Often, models that fail due to underfitting are insufficiently expressive to capture the dynamics of the underlying system.

##### Simplicity:

The second performance criterion is simplicity, with an associated failure mode of feature invention. Invented features are a mirror image of underfitting: while underfit models lack features of the true system, the true system lacks features “invented” by the model. Previous metrics for simplicity quantify how well the ground-truth system can predict the model-inferred features. Models with both optimal simplicity and optimal reconstruction have a 1:1 mapping between the model-inferred latent features and the features of the underlying system.

One particularly insidious form of feature invention occurs when the invented features actually lead to improvements in reconstruction on both training and validation datasets. When ranking DD models by their ability to reconstruct neural activity, it has been shown that models with invented features often *outperform* those without [55]. This suggests that not only is reconstruction insufficient for DD model selection, but relying on reconstruction alone may be actively misleading! While some metrics to quantify model simplicity have been released [39, 46, 55], these methods require access to the ground-truth system and therefore are not applicable to models trained on biological datasets.

We show an example of feature invention in Figure 1G. Though our hypothetical model perfectly captures the true latent activity **z**, it also infers an additional latent dimension ${\hat{z}}^{*}$. This “invented” activity evolves such that when the bit turns off, the state returns to a location that is different from where it began the trial. Alone, reconstruction and simplicity are each insufficient to judge DD model quality, but together they can provide interpretable accounts of DD model performance when external inputs are known.

##### Input Accuracy:

Now we consider the more realistic scenario in which external inputs are unknown and therefore must be inferred alongside the latent dynamics. The third performance criterion is input accuracy, or how well the model-inferred inputs match the true external inputs to the system.

In Figure 1H, we show a hypothetical model that accurately infers latent activity without inventing features, yet fails to capture the true dynamics **f**. The inferred inputs can reconstruct the data without relying on any intrinsic dynamics, while producing exactly the same predictions as the true model! Since both models are equally supported by the evidence, it is impossible to determine which is correct from observations alone – a phenomenon we call dynamical misattribution, an instance of the broader issue of non-identifiability. Because input accuracy cannot be evaluated on most biological datasets, this failure mode is particularly troubling: even when $\hat{z}$ is accurate, $\hat{f}$ can still be a poor estimate of the true **f**.

## 3 Overview of the Computation-through-Dynamics Benchmark

### 3.1 Key Motivation:

Our overall goal is to develop data-driven dynamics models that can distill unobserved latent activity **z**, dynamics **f** , embedding **g**, and external inputs **u** from observable neural activity **y**. The Computation-through-Dynamics Benchmark (CtDB) helps accomplish this goal by addressing two primary shortcomings in data-driven model validation: biological implausibility of existing synthetic systems and insufficiency of existing metrics for assessing model performance. The result is a standardized, extensible codebase that provides actionable feedback on model performance, facilitates comparisons across model architectures, and helps promising model innovations to be communicated more quickly across the community.

**Figure 2:**
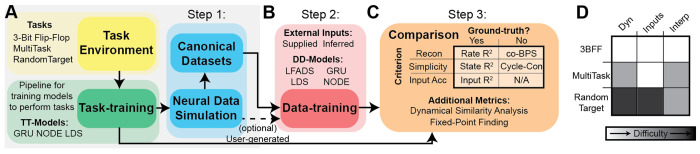
Features of the CtD Benchmark **A**) Dataset generation pipeline- Task Environments (yellow) are taken as input to the task-training pipeline (green), which trains a dynamics model (available models: GRU, NODE, LDS) to perform the task. After training, the TT model is passed to a neural data simulator (cyan), which simulates spiking neural activity with configurable parameters. Users can either generate their own simulated datasets (dashed line) or use the pre-generated canonical datasets (solid line) **B)** Step 2: Data-training pipeline: Train a chosen DD model (available baseline models: LFADS, GRU, LDS, NODE) to reconstruct simulated neural activity, using either supplied or inferred inputs. Trained models are saved for analysis and comparison. **C**) Step 3: Model Comparisons- Top: Table of primary performance metrics, organized by criterion (rows), and need for ground-truth (columns). Bottom: Additional performance metrics/visualization tools. **D**) Included datasets ranked by our subjective assessment of complexity of dynamics, complexity of inputs, and interpretability of tasks from previous research. White, gray, and black denote simple, intermediate, and difficult, respectively.

CtDB streamlines the development process with three simple steps. First, users can choose one of three biologically-realistic datasets simulated from “task-trained” (TT) dynamical models optimized to accomplish specific computational goals (Fig. 2A). Second, users can train new DD models on the provided datasets (Fig. 2B), either by adapting their model to a standardized format, or by exporting simulated datasets to external data-training pipelines. Finally, users can apply CtDB metrics to quantify model performance on reconstruction, simplicity, and input accuracy (Fig. 2C) and compare performance of their model against our included baseline models. We hope that the users will then submit their models to CtDB, allowing others to explore and build upon their promising model innovations! The modular task-training pipeline will also allow us to incorporate user-defined tasks and task-trained models to grow the set of available canonical datasets.

### 3.2 Model developer’s guide to CtDB:

#### Step 1: Select a task:

First, users must select a task to test their data-driven models.

At release, CtDB provides pre-generated datasets for three tasks:

- *Three-Bit Flip-Flop*: Storing a memory state that can be modified by external inputs .
- *MultiTask*: Performing a set of cognitive sub-tasks including category matching, sensory integration, etc.
- *RandomTarget*: Controlling the endpoint of a simplified biomechanical effector to instructed locations.

Each canonical dataset includes everything needed for DD model training and evaluation, including simulated spiking activity and external inputs. We also provide the task-trained models from which the synthetic datasets were generated, allowing users to directly inspect the dynamics of the ground-truth system. Each task was selected to serve a particular role in DD model validation (Fig. 2A): we provide more details on the intended role of each canonical dataset in Section 3.3.

#### Step 2: Train models on canonical datasets:

Within the CtdB data-training pipeline, users can fit models on canonical datasets and easily perform large hyperparameter sweeps (Fig. 2B). We support two training modes: supplied ground-truth external inputs or model-inferred external inputs. In addition to training user-defined models on synthetic data, users have access to a library of baseline models, including standard sequential auto-encoders and LFADS [25]. We define a common interface so that new models can be contributed to CtDB, expanding the stable of baseline models for future developers (Appendix D).

#### Step 3: Compare task-trained and data-driven models:

CtDB provides tools that facilitate interpretation of task-trained and data-driven models, including metrics for quantifying performance on the criteria defined in Section 2.2. Some of the included metrics do not require ground truth and could therefore even be extended to assess models trained on biological datasets (Fig. 2C). CtDB also provides methods for data handling, model inference, fixed-point finding, and model visualization that allow users to quickly and easily assess model performance and iterate during model development (Section 3.4).

#### Optional: Generate new synthetic neural datasets:

For researchers interested in alternative task behaviors or modalities of simulated neural activity, CtDB provides templates for custom tasks and TT model architectures that are compatible with the existing task-training and data simulation pipelines. The simulation pipeline is configurable, allowing users to easily test how changes in neuron count, noise model, or embedding **g** affect the performance of data-driven models (Appendix C) and metrics (Section 3.4).

### 3.3 Task Environments

In contrast with previous validation datasets, CtDB datasets were specifically designed to serve as realistic proxies for biological neural datasets (see Section 2.2). These datasets were simulated from dynamical systems trained to perform specific tasks, defined in CtDB as “task environments”, which provide simulated inputs and objective functions for quantifying TT model performance. Over the course of training, TT models learn dynamics that approximate the input/output mapping of the desired computation. For easy extensibility, task environment objects inherit from the Gymnasium Env class [54].

We include 3 task environments in CtDB, chosen to provide a range in difficulty in 1) complexity of dynamics, 2) complexity of external inputs, and 3) present state of task interpretability (Fig. 2D).

#### Three-Bit Flip-Flop (3BFF):

**Figure 3:**
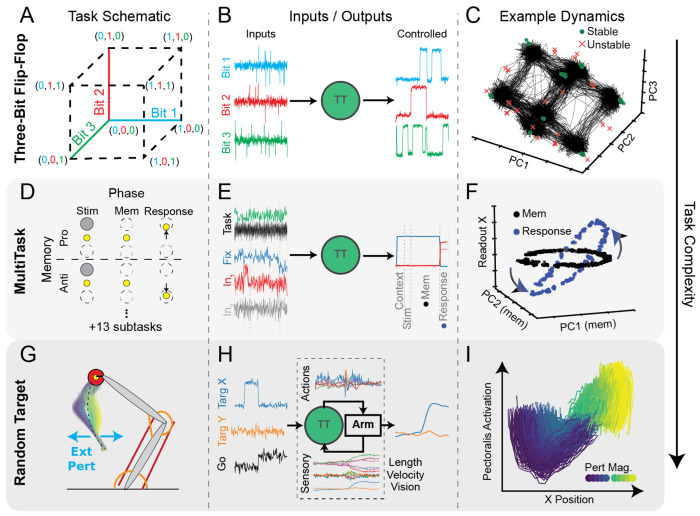
CtDB Datasets have complex and interpretable dynamical features **A**) Schematic of three-bit flip-flop (3BFF) task environment [15]. Three bits encoded according to inputs corresponding to 8 potential memory states. **B**) Inputs (left) and outputs (right) of the task-trained model for an example trial. **C**) Visualization of latent activity (black traces) and fixed points (FP) from canonical 3BFF TT network. Stable FPs (green circles) are found on vertices and unstable FPs (red ×s) along edges of the cube. [23] **D**) Schematic of 2 of 15 tasks in MultiTask environment (MemoryPro, MemoryAnti). Each task has different rules for how to generate outputs. For full details, see [43] **E**) Example of single trial inputs/outputs from canonical MultiTask TT-GRU performing “MemoryPro” task. Task phases indicated by dashed vertical lines. **F**) Example FP architectures during two phases of MemoryPro task (Mem1 (black) and Resp (blue)). Ring of FPs rotates during the response phase into dimensions of the model that affect the output, executing the correct response based on location of activity in the ring attractor. **G**) Schematic of RandomTarget environment [56]. The TT model was trained to control the effector endpoint (yellow circle) to acquire target (red circle) after a go-cue was provided. External forces (cyan) were applied to perturb the hand, random in timing, direction, and magnitude. We show the resulting kinematics of the hand when applying left/right bumps of variable magnitude to the hand during a reach away from the body. **H**) Example of single trial inputs/outputs for canonical TT-GRU performing RandomTarget. As RandomTarget is coupled, actions (muscle commands) and sensory signals (muscle kinematics, visual inputs) are transmitted between the TT-GRU and the arm. **I**) Latent activity in a behaviorally-relevant plane of canonical TT-GRU when responding to perturbation in G. X-axis: Dimension of TT latent activity with strongest linear relationship to x-position of hand. Y-axis: magnitude of projection of TT latent activity onto Pectoralis motor-potent dimension. Bump magnitude (left:blue/right:green-yellow) shown in inset color-map.

3BFF is a 3-bit memory task with the goal of remembering the sign of the last input pulse on each of three noisy input channels (Fig. 3A). We chose to include 3BFF in CtDB because it has 1) simple dynamics, 2) simple inputs (Fig. 3B), and 3) is well characterized by previous research [15]. TT models performing 3BFF typically exhibit low-dimensional activity organized into a cube, with stable fixed points at the vertices and unstable fixed points along the edges (Fig. 3C).

Intuitively, RNNs trained to perform 3BFF use stable fixed points to remember the current memory state, and unstable fixed points to guide input-driven transitions between memory states. With low-dimensional activity and well-characterized dynamics, synthetic 3BFF datasets are intended to provide data-driven model developers with clear and unambiguous feedback about model performance. As such, we consider 3BFF to be the “entry-level” task, and recommend it as the first task on which data-driven model performance is evaluated.

#### MultiTask:

**Figure 4:**
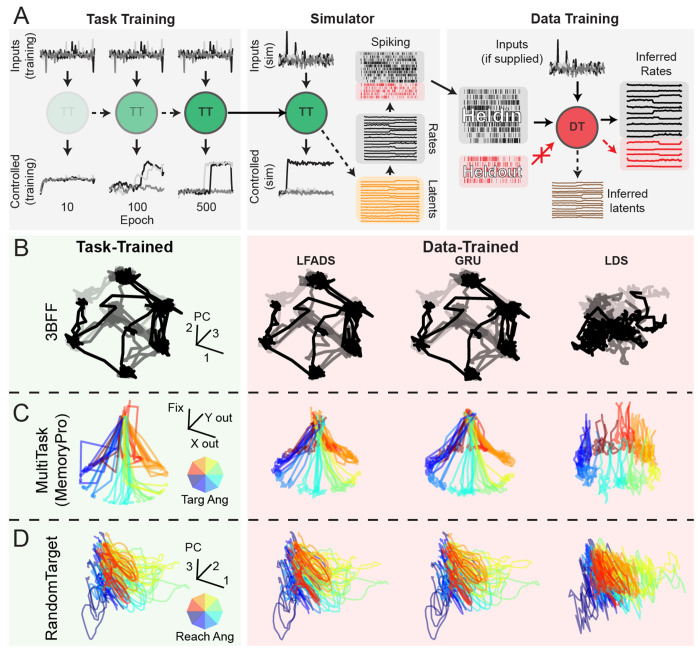
Task-trained and data-driven modeling pipeline, and example inferred latent activity for canonical datasets: **A)** Schematic of task-training, simulation, and data-training pipelines: (left) Task-training: model inputs converted to control signal by TT model dynamics. Over training epochs, TT models learn to generate control output that accomplishes the task. (middle) Fully-trained model provided as input to the simulator. Simulator was used to sample latent activity from the TT model, convert to rates, and simulate spiking using a Poisson noise process. (right) Simulated spiking split into held-in and held-out sets. Held-in spiking is fed as input to the DD model, which is trained to infer rates for both held-in and held-out neurons. Hidden activity of the DD model is referred to as inferred latent activity. **B-D)** Example ground-truth latent activity (left column, green, without noise in input channels for improved visualization) and latent activity inferred by DD models (from left to right, LFADS, GRU, LDS) for three canonical datasets. DD model inferred latent activity was aligned with TT latent activity using an optimal affine transformation. **B)** Latent activity (top 3 PCs) for three representative trials (indicated by opacity) for 3BFF canonical dataset. **C)** Latent activity for representative trials of MemoryPro subtask for MultiTask canonical dataset. Color of each trace indicates the angle of the correct output response (inset color wheel). Latent activity was projected onto a 3D subspace defined by response 1 (x-axis), response 2 (y-axis), and fixation (z-axis) output dimensions. For DD models, we transformed the inferred latents to the space of the ground-truth latents via an optimal affine transformation, then projected through the ground-truth output mapping. **D)** Example inferred latent activity (top 3 PCs) for representative trials for RandomTarget canonical dataset. Color indicates the reach angle for each trial.

MultiTask consists of 15 distinct cognitive tasks and has been used to investigate shared representations and learning in task-trained networks (Fig. 4D) [33, 43, 44]. Networks trained on MultiTask have 1) moderately complex dynamics, 2) simple, but relatively high-dimensional inputs, and 3) shared dynamical structures that provide a foothold for interpreting how the computations are performed.

Inputs to MultiTask include a one-of-15 (one-hot) input set that indicates which task is engaged on each trial, 4 noisy sensory input channels, and one fixation input (Fig. 4E). For each task, sensory inputs must be transformed into 3-dimensional outputs that follow a task-specific rule. Each task has piecewise inputs that change abruptly across phases– e.g., input presentation, delay period, etc. We show simplified versions of 2 example tasks and their phases in 3D (more details in Appendix B.1.2 and [33, 43]).

Models trained to perform MultiTask learn “dynamical motifs”: shared structures that are reused across multiple tasks [43]. Because MultiTask networks receive piecewise constant external inputs, fixed point (FP) analysis can be used to provide an interpretable account of how the dynamics change across task phases. Previous research studying MultiTask has provided a clear demonstration of how input-dependent changes in FP structure can shed light on how computation is organized in a dynamical system.

To demonstrate how the dynamics of the MultiTask network can provide interpretable accounts of the computation, we analyzed the FP structure of a Multitask TT model performing the MemoryPro task (Fig. 3F). As shown previously [38], trained models exhibit a ring attractor of stable FPs that are used to memorize a continuous circular input. When the model was prompted to produce an output corresponding to the input it had seen previously [43], the FP ring rotated into a set of output dimensions that generated the correct response. With its complex and shared dynamical features and its piecewise constant inputs, MultiTask represents the current limits of our ability to interpret task-trained dynamics models, and therefore a challenging but tractable system for data-driven model validation.

#### RandomTarget:

The RandomTarget task was inspired by a common experimental paradigm in motor neuroscience [4, 26], planar arm reaching. RandomTarget involves controlling a 2 degree-of-freedom arm model actuated by 6 Hill-type muscles, simulated using the MotorNet musculoskeletal modeling package [56]. TT models trained on RandomTarget must learn to produce muscle output that moves the arm’s endpoint to target locations sampled randomly from within the arm’s range of motion, while also receiving and correcting for intermittent bump perturbations applied to the hand (Fig. 3G).

Models trained to perform this rich motor control task have 1) complex dynamics with 2) high-dimensional time-varying inputs, and 3) underlying computations that are not yet well-understood. The model receives both sensory (muscle lengths and velocities, hand endpoint position) and contextual (target position, go cue) inputs, and generates efferent commands that influence the force generated by each muscle. In contrast with previous tasks, this synthetic system is coupled to the environment, meaning that the inputs that it receives are affected by the motor commands it generates (and vice versa, see Fig. 3H)). As the inputs are constantly changing, this can make direct interpretation of the dynamics very difficult.

To find a signature of dynamical computations underlying movement corrections, we trained a model on RandomTarget and tested how it learned to correct for left/right perturbations in the middle of a reach away from the body (Fig. 3G, colored trajectories). To visualize how sensory information is transformed to produce corrective muscle commands, we projected the TT model’s activity onto the plane defined by the pectoralis muscle activation (responsible for shoulder flexion) and the endpoint *x*-position. We found a stereotyped rotational pattern in this plane that varied smoothly with the perturbation magnitude and direction (Fig. 3I, color gradient). Interestingly, TT model dynamics have learned to perform goal-directed sensory-motor transformations that correct for perturbations to the desired hand trajectory; inspecting this activity reveals an interpretable structure that seems to resemble a feedback control system [38]. We expect that there are many other interesting features to be discovered in the dynamics of TT models trained on RandomTarget.

Given the combination of task complexity, time-varying inputs, and lack of prior understanding of the computational structures involved, RandomTarget is perhaps the most challenging dataset currently included in CtDB. As biological circuits also receive complex, time-varying inputs, RandomTarget is also arguably most similar to datasets that might be collected experimentally.

### 3.4 Metrics and Visualizations

Recent research has shown that reconstruction can be an unreliable indicator of model quality [46]. CtDB includes new metrics that provide a more holistic account of model performance on key criteria of reconstruction, simplicity, and input accuracy. Some of these metrics (Rate *R*^2^, State *R*^2^, Input *R*^2^) require access to ground-truth, while others (co-smoothing bits-per-spike and cycle-consistency) can be applied even when the ground-truth is unknown.

Also included in CtDB is a previously released method called Dynamical Similarity Analysis (DSA [51]), which quantifies aspects of both reconstruction and simplicity. We show in Section 4 how these metrics can be used to identify common failure modes of DD models, and how they can be used to guide model and hyperparameter selection. We used held-out validation datasets for all metrics and visualizations to prevent overfitting from affecting our interpretations of DD model quality.

#### 3.4.1 Metrics comparing DD models to ground-truth

##### Rate *R*^2^ :

Rate *R*^2^ measures a DD model’s ability to capture features of the true system (i.e., firing rates). Rate *R*^2^ is obtained by finding the coefficient of determination between the true and predicted firing rates of each neuron in the dataset, yielding *N R*^2^ values, which we summarize with the variance-weighted mean across all neurons. By weighting by each neuron’s firing rate variance, Rate *R*^2^ is not artificially deflated by poorly predicted neurons that are only weakly modulated by the task. A Rate *R*^2^ value of 1 indicates perfect reconstruction of the underlying rates for all neurons. As Rate *R*^2^ requires access to ground-truth neural firing rates, it is not typically applicable for DD models trained on biological datasets.


(1) $$R_{\text{Rate}}^{2}=\frac{\sum_{i=1}^{N}\text{Var}\left(N_{i}\right)R^{2}\left(N_{i},{\hat{N}}_{i}\right)}{\sum_{i=1}^{N}\text{Var}\left(N_{i}\right)}$$

##### State *R*^2^ :

The possibility of invented features means that a DD model’s reconstruction is an unreliable indicator of accurately inferred dynamics (see Fig. 1G). The first CtDB metric for quantifying model simplicity is called State *R*^2^ [46]. State *R*^2^ assesses the degree to which the inferred latent activity contains features that cannot be linearly explained by the ground-truth latent activity. State *R*^2^ is computed by fitting an affine transformation from the true hidden unit activity of the TT model ($z\in {\mathbb{R}}^{d_{z}}$) to the activity inferred by the DD model ($\hat{z}\in {\mathbb{R}}^{d_{\hat{z}}}$), and then computing the variance-weighted coefficient of determination. As State *R*^2^ requires access to ground-truth latent activity, it is not typically applicable for DD models trained on biological datasets.


(2) $$R_{\text{State}}^{2}=\frac{\sum_{i=1}^{d_{\hat{Z}}}\text{Var}\left({\hat{Z}}_{i}\right)R^{2}\left({\hat{Z}}_{i},AZ_{i}+d\right)}{\sum_{i=1}^{d_{\hat{Z}}}\text{Var}\left({\hat{Z}}_{i}\right)}$$

##### Input *R*^2^

As shown in Figure 1H, accurately inferred inputs are critical to ensure that a DD model’s inferred dynamics are trustworthy. We provide a metric called Input *R*^2^ that assesses the accuracy of these inferred inputs. Similar to State *R*^2^, Input *R*^2^ is calculated by fitting an affine transformation from inferred inputs ($\hat{U}\in {\mathbb{R}}^{d_{\hat{U}}}$) to true inputs ($U\in {\mathbb{R}}^{d_{U}}$), then computing the variance-weighted coefficient of determination across input dimensions. As Input *R*^2^ requires access to ground-truth external inputs, it is not typically applicable for DD models trained on biological datasets.


(3) $$R_{\text{Input}}^{2}=\frac{\sum_{i=1}^{d_{U}}\text{Var}\left(U_{i}\right)R^{2}\left(U_{i},C{\hat{U}}_{i}+d\right)}{\sum_{i=1}^{d_{U}}\text{Var}\left(U_{i}\right)}$$

#### 3.4.2 Metrics assessing DD models without ground-truth

Because dynamics and external inputs are often unobservable for biological systems, it is important that we develop metrics that can be applied even when we are unable to compare against ground-truth. In this section, we describe two metrics, co-BPS and cycle-consistency, that quantify DD model performance on the criteria of reconstruction and simplicity.

##### Co-Smoothing Bits-per-Spike:

Co-smoothing bits-per-spike (co-BPS) is a previously released method to assess DD model reconstruction [45]. co-BPS quantifies how well spiking activity in a set of held-out neurons can be predicted by models that only have access to a set of held-in neurons. Assuming a Poisson observation model, co-BPS will be positive if the predicted activity of held-out neurons is more informative than their mean firing rate. While this metric makes some assumptions about the observation model, it does not require access to ground truth firing rates to compute. co-BPS is calculated as:

$$\text{co-bps}=\frac{1}{y_{\text{sp}}\log2}\left(L\left(\hat{n};{\hat{y}}_{i,t}\right)-L\left({\bar{n}}_{i};{\hat{y}}_{i,t}\right)\right)$$

where $y_{\text{sp}}$ is the total number of spikes from the held-out neurons, L is the negative log-likelihood function, $\hat{n}$ represents the model-predicted firing rates, ${\bar{n}}_{i}$ is the mean firing rate of neuron i, and ${\hat{y}}_{i,t}$ are the observed spike counts for neuron i at time t.

##### Cycle-Consistency:

To ensure that DD models trained on biological datasets don’t suffer from invented features, we need a method for assessing simplicity of DD models that doesn’t require access to ground-truth latent activity. Our method, called cycle-consistency, provides an indirect estimate of simplicity by interrogating the relationship between inferred latent activity and inferred firing rates.

By definition, DD models suffer a penalty to reconstruction if invented features are directly visible in predicted neural activity. For this reason, DD models are incentivized to invent features that do not affect neural predictions. This is only possible if the model learns an embedding $\hat{g}$ from latent activity to neural predictions that is *non-injective* [55]. Non-injective embeddings allow some changes in latent activity to produce no change in their output. A common example of a non-injective embedding is a linear embedding with a null-space; activity can change in dimensions of the null space without affecting the predicted neural activity (as seen in Fig. 1G).

Cycle-consistency quantifies the extent of non-injectivity of the inferred embedding $\hat{g}$ and uses it as an indirect measure of feature invention. Models with high cycle-consistency have few features in their inferred latent activity that are not reflected in the predicted neural activity – i.e., they have few invented features.

Mathematically, cycle-consistency tests the ability of a linear model to re-generate inferred latents $\hat{z}$ from inferred neural activity $\hat{n}$. Cycle-consistency is computed by 1) performing principal components analysis (PCA) on inferred latents and inferred log-rates to sort dimensions by their explained variance, 2) fitting a linear readout from inferred latent activity to the inferred log-rates, 3) applying singular value thresholding (by default, singular values associated with <1% of the total variance) to this mapping to eliminate dimensions that are effectively null [7], and 4) using the pseudo-inverse of this linear mapping to re-generate inferred latents. We compute the variance-weighted *R*^2^ score between inferred latents $\hat{z}$ and re-generated latents $\tilde{z}$:

$$R_{\text{Cycle}}^{2}=1-\frac{\sum_{i}w_{i}{\left({\hat{z}}_{i}-{\tilde{z}}_{i}\right)}^{2}}{\sum_{i}w_{i}{\left({\hat{z}}_{i}-\bar{z}\right)}^{2}}$$

Here, ${\hat{z}}_{i}$ are the inferred latent variables rotated by PCA, ${\tilde{z}}_{i}$ are the re-generated latent variables obtained from the pseudo-inverse mapping, $\bar{z}$ is the mean of the inferred latent variables, and $w_{i}$ are weights proportional to the variance of each latent dimension. Because this metric is computed using only the inferred variables, it can be calculated without access to the ground-truth system.

#### 3.4.3 Additional Metrics and Visualization

##### Dynamical Similarity Analysis (DSA) :

DSA [51] is a nonlinear similarity metric that compares the spatiotemporal structure of two dynamical systems. Applied to CtDB, DSA measures whether DD models reconstruct the core dynamic features of their TT model. More specifically, DSA captures whether these features (e.g. fixed points) are aligned in a one-to-one fashion and the DD model has neither invented superfluous features nor deleted relevant ones. DSA has three components (further details are in Appendix E.3): first, the method lifts the data into a higher dimensional space using kernels or delay embeddings [17, 28]. Second, a linear system is fit to each embedding, resulting in dynamics matrices $A_{x},A_{y}$. Finally, these systems are compared using an extension of Procrustes Analysis [42]. In this paper, we used the angular form of the distance, which scales from 0 (most similar) to π/2 (most dissimilar). In practice, however the full range is not always used, and so relative distances are typically emphasized over absolute.


(4) $$\text{DSA}\left(A_{x},A_{y}\right)=\underset{C\in O\left(n\right)}{\min}{\left\|A_{x}-CA_{y}C^{-1}\right\|}_{F}$$

##### Fixed-Point Visualization :

In addition to the quantitative metrics, CtDB provides tools for qualitatively assessing the true and inferred dynamics through their fixed point structures. Fixed points define regions of state space where the dynamics are sufficiently slow to permit linear approximation. The arrangement and stability of these fixed points provide key insights into the local behavior of the system. To find fixed points, we find locations in the model’s state space ($Z\in {\mathbb{R}}^{d_{z}}$) that minimize the magnitude of the kinetic energy of the system ($\text{Δ}h^{2}\sim q$). CtDB includes an extension of a previously-released fixed point finding toolkit [23] for use with both TT- and DD- models. Since input changes can alter fixed point structure [43, 31], our modified fixed point finder accepts user-defined static inputs, allowing researchers to visualize how fixed point structures change as inputs change.

## 4 Results

### 4.1 Canonical datasets provide a library of biologically-plausible dynamical systems

#### Task-training and dataset simulation pipeline:

The data-simulation process began by using a CtDB task environment to generate a training dataset containing external inputs, target outputs, and additional information needed for training (see section B.1 for more detail). These datasets were used to train a TT dynamics model to produce outputs that minimize the objective function defined by the task. Over the course of training, task-trained (TT) models learned to transform these inputs into outputs that accomplish the task (Fig. 4A, left). By the end of training, models had learned latent dynamics that successfully performed the computation defined by each task environment.

Once task-training was complete, synthetic neural datasets were simulated from the hidden unit activity of the TT models (Fig. 4A, middle, see Appendix B for more details). Trials from the task-training dataset were fed to the TT model and the resulting hidden activity for each trial was recorded. *N* dimensions of hidden activity were sampled without replacement, scaled, and used as the rate parameter of a Poisson process. This generated a dataset of spiking activity from *N* simulated neurons, which was then split into held-in and held-out neuron sets (see Appendix C.1 for more details). We trained DD models to reconstruct held-in and held-out neural activity using only held-in neural activity (Fig. 4A, right).

#### Key observations from canonical datasets:

To provide some intuition for how common DD models perform on the canonical datasets, we visualized the ground-truth and inferred latent activity for the three tasks included in CtDB: 3-Bit Flip-Flop (3BFF), MultiTask, and RandomTarget. We provide descriptions of the three baseline DD models in Section D.3.

##### 3-Bit Flip-Flop (3BFF):

For 3BFF (Figure 4B), we found that LFADS and GRU models inferred latent activity that closely resembled the top three principal components (PCs) of the ground-truth latent dynamics. In contrast, we found that the limited computational capacity of LDS made it unable to model even the relatively simple dynamics that are found in 3BFF. In other words, LDS suffers from the failure mode of *underfitting* described in Section 2.2.

##### MultiTask:

In the MultiTask dataset (Figure 4C), we visualized the behavior of our DD models by projecting the inferred latent activity from MemoryPro trials onto three output dimensions (Fixation, X output, Y output) for each model.

We found that both GRU and LFADS models qualitatively captured the structure of the ground-truth latent activity in this 3D subspace. However, the LDS model had significant errors in its projection onto output dimensions during the memory period. This suggests that LDS was unable to gate its response appropriately, which would result in premature output generation. Similar to 3BFF, these results indicate that LDS lacks computational capacity, and as a result also underfits the dynamics of the MultiTask dataset.

##### RandomTarget:

Given its complex time-varying inputs, RandomTarget (Figure 4D) is the most difficult dataset to interpret. We examined the top three PCs of latent activity, color-coded by reach direction and found that all three models seemed to capture the high-level structure of the ground-truth latent activity. As these DD models were provided ground-truth external inputs during training and inference, comprising time-varying muscle lengths/velocities, vision, and goal/timing information, it is unclear how much of the complexity of the inferred latent activity is inherited from external inputs rather than generated via the model’s intrinsic dynamics. The difficult task of disentangling intrinsic dynamics from external inputs makes RandomTarget a highly realistic and challenging addition to CtDB [38].

### 4.2 Reconstruction and simplicity metrics guide model selection and avoid common failure modes

Next, we sought to demonstrate that CtDB metrics can provide actionable feedback about how to improve DD model performance. We trained a set of DD Neural ODE (NODE) models to reconstruct synthetic neural spiking activity generated from our canonical 3BFF TT model [22] see Appendix D.3 for more detail). For NODE models, increasing the dimensionality has the effect of increasing the expressivity of the dynamics; for this reason, we trained 5 randomly initialized DD NODE models for each hidden dimensionality $\hat{D}\in [3,5,8,16,32,64]$ (30 models in total), supplying all models with ground-truth external inputs. This allows us to investigate how different levels of model capacity can lead to distinct failure modes.

**Figure 5:**
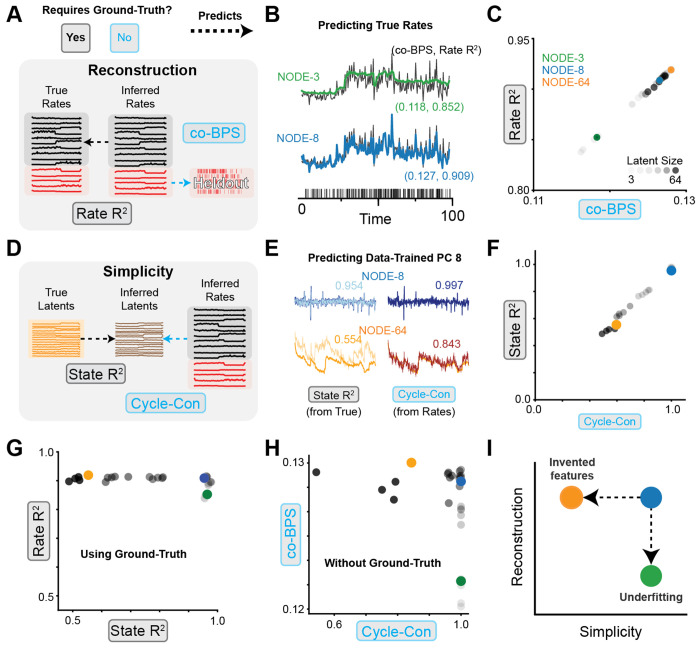
Reconstruction and simplicity metrics for data-driven modeling of 3BFF dataset **A)** Schematic of reconstruction metrics: Rate *R*^2^ measures prediction accuracy of inferred rates vs. true rates. co-BPS measures accuracy of held-out spiking predictions. **B**) Single-trial predicted firing rate of one held-out neuron from models NODE-3 (3 hidden unit NODE, green) and NODE-8 (8 hidden unit NODE, blue). Performance indicated by (Rate *R*^2^, co-BPS) inset. **C)** Scatter plot of Rate *R*^2^ vs. co-BPS for NODE models ranging from 3 to 64 hidden units. Higher opacity indicates larger hidden size. **D)** Schematic of simplicity metrics: State *R*^2^ measures prediction accuracy of inferred latents from true latents, cycle-con measures accuracy of inferred latents from inferred rates. **E)** Inferred latent activity PC8 (blue = NODE-8, orange = NODE-64), and linear prediction from true latents (left, State *R*^2^) or inferred rates (right, cycle-con). **F)** Scatter plot of State *R*^2^ vs. cycle-con for same models in panel C, indicated by colored markers. **G)** Scatter plot of Rate *R*^2^ vs. State *R*^2^, measuring reconstruction and simplicity with access to ground-truth. **H)** Scatter plot of co-BPS vs. cycle-con, measuring reconstruction and simplicity without access to ground truth. **I)** Schematic of underfitting (green, NODE-3) and invented features (orange, NODE-64) failure modes, for comparison with panels G, H.

First, we quantified each model’s reconstruction using Rate *R*^2^ and co-BPS, as described in Section 3.4 (Fig. 5A). Inspecting the inferred firing rate of a single held-out neuron, we found that while DD NODE with 3 dimensions (NODE-3) was able to capture some aspects of the true firing rate, its reconstruction was substantially worse than a DD NODE with 8 hidden units (NODE-8, Fig. 5B, green, blue, respectively). Models with a very small number of hidden units apparently had insufficient capacity, and therefore suffered from *underfitting*.

To confirm that our metric for quantifying reconstruction without access to ground-truth firing rates (co-BPS) gave similar results to our ground-truth metric (Rate *R*^2^), we plotted the performance of our 30 models on these two metrics and found a strong linear relationship between Rate *R*^2^ and co-BPS (Fig. 5C). The co-smoothing metric used to quantify reconstruction in previous benchmarks [45] seems to accurately capture reconstruction, even in the absence of ground-truth.

Next, we assessed the simplicity of each model to determine the extent to which they had invented features (Fig. 5D). To do this, we visualized the inferred latent activity in a low-variance dimension (8th principal component) for two DD models (Fig. 5E, blue: NODE-8, orange: NODE-64), along with linear predictions of this dimension from the true latents (State *R*^2^, left column) and inferred rates (Cycle-Con, right column). We found that the 8th PC of NODE-64 was predicted poorly compared to the 8th PC of NODE-8. The presence of features in the inferred latent activity that cannot be predicted from the ground-truth suggests that the more expressive models tended to invent features that didn’t exist in the underlying system.

To confirm that our metric for quantifying simplicity without access to ground-truth latents (cycle-consistency) gave similar results to our ground-truth simplicity metric (State *R*^2^), we plotted the performance of our 30 models on these metrics and, like our reconstruction metrics, found a strong linear relationship (Fig. 5F). Additionally, we found that as the NODE hidden size increased, there was a consistent drop in the simplicity for both metrics, giving further evidence that more expressive models may suffer from the failure mode of *feature invention*.

We then plotted our metrics for reconstruction and simplicity, showing metrics which rely on access to ground-truth (Fig. 5G) and metrics that do not (Fig. 5H). Both sets of metrics qualitatively recapitulate the failure modes described by our performance criteria presented in Figure 1. Models with insufficient expressivity suffered from underfitting, while models that were too expressive invented features (Fig. 5I).

Because State *R*^2^ and cycle-consistency are linear methods, nonlinearities in the mapping between two topologically identical systems could lead these metrics to diagnose a model with invented features. In contrast, DSA should be insensitive to nonlinear distortions of the inferred activity. In Supp. Fig. 12, we show that DSA may be able to ignore such trivial non-linearities. However, it is still unclear whether these geometric differences can be safely ignored, so CtDB includes both DSA and simpler linear metrics as complementary measures of model similarity.

### 4.3 Input *R*^2^ helps diagnose models that fail by dynamical misattribution

While the above experiments consider situations where ground-truth inputs are known, this is very rarely the case in biological neural datasets. Another metric included in CtDB, Input *R*^2^, quantifies the ability of a model to accurately infer unseen external inputs.

**Figure 6:**
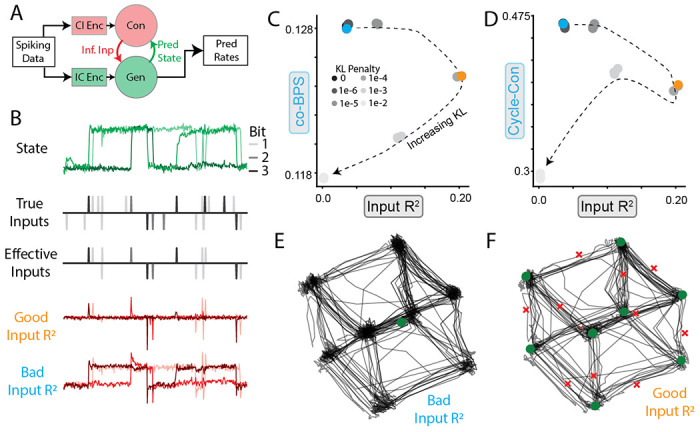
Inferred inputs affect inferred dynamics **A**) Schematic of common input inference procedure for DD models. IC Enc, CI Enc denote Initial Condition encoder, Controller Input encoder, respectively. Ideally, the generator only models the intrinsic dynamics of the circuit and the controller only predicts inputs when inputs are actually present. **B**. Example activity from a single trial of 3BFF task. Shading indicates bit number. Top row: Output of TT-GRU. Rows 2-3: True and effective inputs (inputs that are expected to affect the state). Rows 4-5: Inferred inputs for two example models with “good” (orange) and “bad” (cyan) input inference. **C**) Scatter plot of co-BPS vs. Input *R*^2^ for models with increasing KL penalty (indicated by shading/ dashed line). Models depicted in B shown in orange/cyan. **D**) Same as C, except cycle-consistency vs. Input *R*^2^. **E**) Inferred FP architecture of model colored in cyan in (C). Stable (unstable) FPs are depicted as green circles (red ×s). **F**) Same as E, except for orange model in (C).

A common architecture for input inference (originally introduced in LFADS [25]) is shown in Figure 6A. Neural activity is provided to two separate modules, the controller and the generator, which produce the inferred inputs and underlying dynamics respectively. Input inference is in general an ill-posed problem because observations alone are insufficient to distinguish a purely input-driven system from a purely autonomous one. Thus, the form of inferred inputs is heavily influenced by model hyperparameters. In the case of LFADS, two critical choices are the prior distribution of the inferred inputs and the magnitude of the penalty for divergence from that prior.

To validate that using Input *R*^2^ to select hyperparameters leads to accurate inferred inputs and dynamics, we fit multiple LFADS models with different weights on the prior distribution of inferred inputs (Student’s *t*-distribution with *df* = 5, which promotes sparse inputs) to data from the 3BFF canonical dataset. We compared the inferred inputs to both the true inputs and the “effective” inputs (i.e. inputs that would change the sign of the bit, as opposed to redundantly indicating the current sign, Fig. 6B, top three rows). Some models inferred inputs that qualitatively matched the effective inputs, while other models’ inferred inputs resembled the state of the network.

Plotting the models’ co-BPS and cycle-consistency against the Input *R*^2^ (Fig. 6C,D) demonstrated that many models with high co-bps and cycle-consistency had extremely poor input inference. As the KL penalty increased, Input *R*^2^ increased until the KL penalty became high enough that the model began to suffer from underfitting (Fig. 6C). Interestingly, we found that models with both high and low Input *R*^2^ could find inferred activity that was almost identical to the true system (Fig. 6E,F, black traces). However, FP analysis revealed that only models with high Input *R*^2^ reproduced the expected FP structure (Fig. 6E,F, ∘, × markers), suggesting that the model with low KL penalty suffered from the failure mode of *dynamical misattribution* (Fig. 1H).

These results provide a striking demonstration that reconstruction and simplicity alone may be misleading when input inference is considered. Reconstruction, simplicity, and input accuracy each provides a piece of the puzzle for selecting data-driven models that can accurately infer dynamics from recorded neural activity.

## 5 Discussion, Limitations, and Future Directions

The Computation-through-Dynamics Benchmark (CtDB) represents a significant step towards evaluating goal-directed neural computations through neural dynamics modeling. By offering standardized datasets and interpretable metrics, CtDB provides researchers with tools to validate data-driven (DD) models, even when both dynamics and inputs must be inferred. Its modular and extensible design invites contributions from the community, to promote continued evolution to address emerging challenges.

Despite its strengths, CtDB faces limitations due to our incomplete understanding of neural dynamics. Current approaches assume that rate coding is sufficient to describe neural dynamics, which may be ignoring important intracellular and neuromodulatory processes. Future extensions could incorporate temporal coding and diffuse neuromodulatory influences to test these assumptions and allow CtDB to be useful to an even wider audience.

Another limitation is the assumption of linear embeddings of latent dynamics into neural activity. Most of the included metrics rely on assumptions of linearity. While CtDB includes the non-linear DSA method [51], further work is needed to assess how deviations from linearity affect model performance and to develop additional metrics that are robust to these deviations [18, 37].

Input inference remains a major challenge. Although metrics like co-BPS and cycle-consistency estimate reconstruction and simplicity without ground-truth, CtDB lacks an analogous metric for input accuracy. Simulated perturbations and new metrics for quantifying input inference accuracy could help address this issue. We consider this to be a major focus of future work in this area.

Looking forward, CtDB’s extensibility offers pathways for addressing these limitations. Contributions such as richer datasets, novel metrics, and improved nonlinear methods can enhance the utility of the benchmark. With continued community collaboration, CtDB can drive innovations that deepen our understanding of neural computation and help bridge the gap between models and biological systems. In summary, CtDB not only provides a foundation for evaluating neural dynamics models but also a robust launch-pad for broader investigations into neural computation. As the field advances, CtDB has the potential to play a key role in shaping tools and frameworks that uncover fundamental principles of brain function.

## Figures and Tables

**Table 1:** **Author contributions.** Shaded cells indicate areas of significant involvement. **Concept** includes ideation and experimental design; **Implementation** includes coding, model development, debugging, and/or website creation; **Figures** includes figure creation and data visualizations; **Writing** includes manuscript drafting, revising, and editing; **Datasets** includes guidance on synthetic dataset creation and critical external code packages; **Strategy** indicates broad scientific and conceptual guidance on overall project goals.

Author	Concept	Implementation	Figures	Writing	Datasets	Strategy
**Chris Versteeg**						
**Jonathan D. McCart**						
**Mitchell Ostrow**						
**David M. Zoltowski**						
**Clayton B. Washington**						
**Laura Driscoll**						
**Jonathan A. Michaels**						
**Olivier Codol**						
**Scott W. Linderman**						
**David Sussillo**						
**Chethan Pandarinath**						

**Figure 7:**
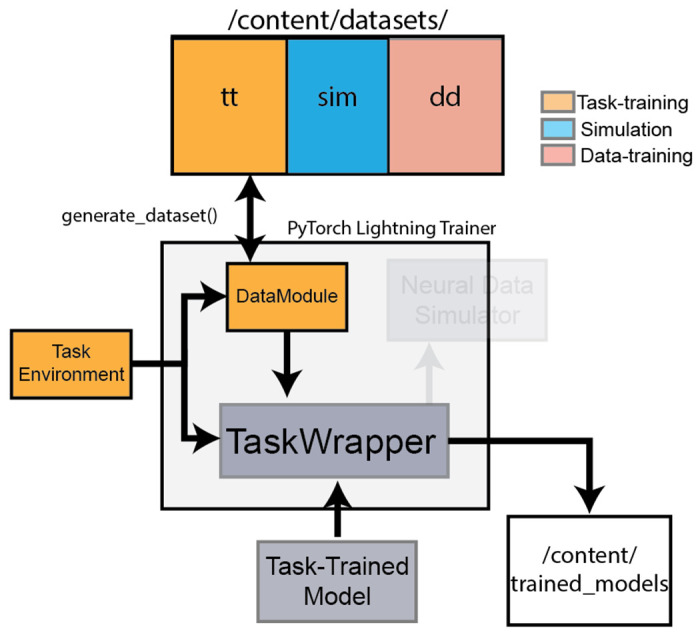
Schematic of task-training pipeline. Here we show the general procedure for running task training with the structure of the objects that appear in the code, and their interactions, shown graphically. First, the data that define the inputs/outputs of a task must be generated. If the dataset has already been generated, it can be found in /content/datasets/tt; otherwise, the datamodule will call the method generate_dataset() from the TaskEnvironment. The task data will then be split into training and validation datasets by the DataModule. In the case of coupled environments that require feedback at each step (e.g. RandomTarget), the TaskEnvironment is also loaded. CtDB uses the TaskWrapper to coordinate training and saving, so each of these objects are passed together to the TaskWrapper which runs the task-training pipeline described in Section F. When training is complete, the DataModule, TaskEnvironment and task-trained model are saved to /content/trained_models.

**Figure 8:**
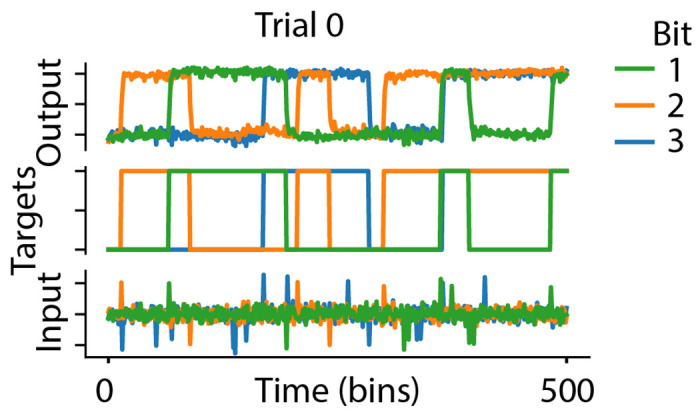
Example trial for 3BFF

**Figure 9:**
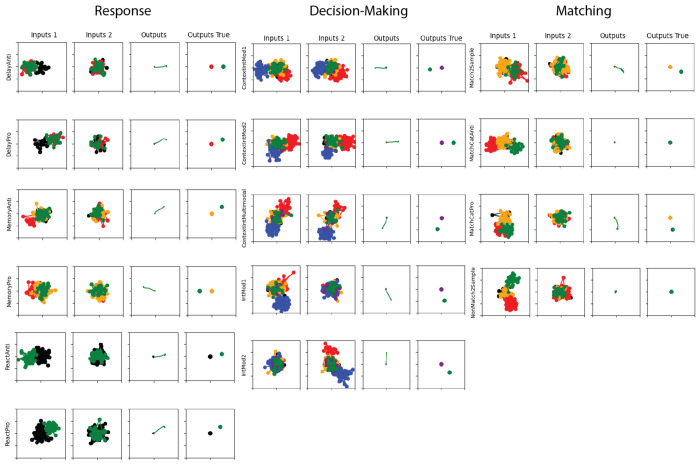
Example trials for MultiTask We show one example trial for each task in MultiTask, organized in columns by the trial category. Each trial has four subplots, with the task name indicated to the left of each row. Each subplot shows a 2D signal over time as a scatter plot, color coded by the task phase. Left two plots indicate the inputs to stimulus modality 1,2, respectively. Third plot indicates the model output on its two response channels. Rightmost plot indicates the targeted output.

**Figure 10:**
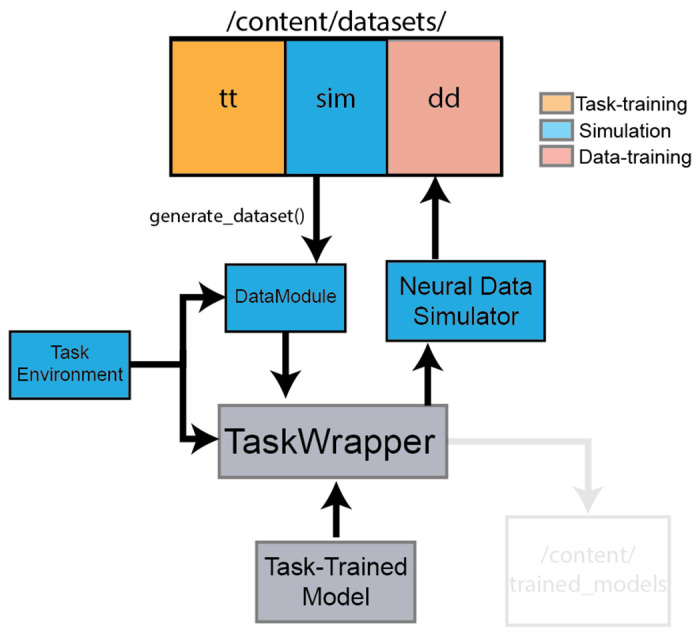
Neural Data Simulation Pipeline. We use a separate directory in the case of task data used for generating the simulated neural data (sim) compared to running task training (tt). This is because there are often differences in how the task is run to ensure stable and efficient training that are not necessary or desirable to consider as part of the trial structure in the simulated data (see Section B.1.3 and Table 7 for intuition in the case of the RandomTarget task.) Similar to the task-training pipeline, the task data is loaded into a DataModule, this time not for training, but instead as a means of curating the trials for passing them through the trained model to obtain the hidden unit activations. Also parallel to the pipeline in Figure 7, the TaskEnvironent is loaded in the case of coupled environments to enable feedback at each step. Once the DataModule and TaskEnvironment are prepared, they are passed to the TaskWrapper which coordinates the evaluation of the task-trained model on each of the trials of the task data from sim. Once all trials have been processed by the task-trained model, the hidden unit activations are used by the Neural Data Simulator to produce the simulated neural spiking activity for the task (more details in C.1). The simulated data is then saved to dd, where it can be accessed later when running the data training pipeline (see Section D and Figure 11.)

**Figure 11:**
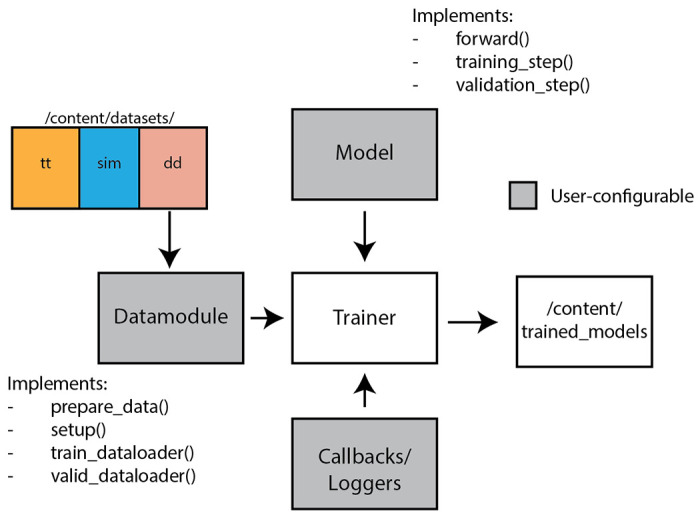
High-level schematic of data-training pipeline. The simulated neural data file is loaded from dd by the prepare_data() and setup() methods of the DataModule, and packaged into training and validation dataloaders. These dataloaders are used to train the data-driven model, an implementation of a LightningModule. The Trainer also accepts user-defined callbacks and loggers. The Trainer object automates handling of the training and evaluation loops, as well as model optimization. After training is complete, the data-driven model and the corresponding datamodule are saved to /content/trained_models.

**Figure 12:**
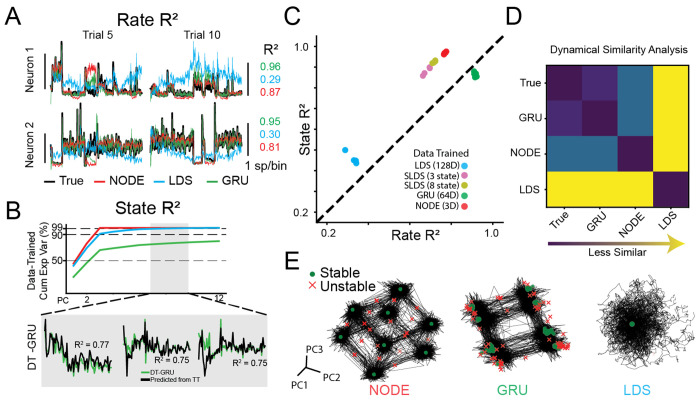
Demonstrations of SLDS, DSA on CtDB 3BFF dataset: **A)** Example single neuron firing rates (black) and predicted firing rates for two example neurons/trials (rows/columns respectively) for three example models (NODE: red, LDS: cyan, GRU: green). *R*^2^ of neural prediction shown at right. Scale bars indicate 1 spike/bin. **B)** (top) Cumulative variance explained as function of PC for each DD model (colors as in A). High-D GRU models require >12 dimensions to reach 90% variance. (bottom) Predictions of the 8,9,10th PC of inferred latent activity from the ground-truth latent activity of the TT model. *R*^2^ for each dimension shown in panel. **C)** Scatter plot of 5 randomly initialized models, color coded by DD model (see legend). **D)** DSA applied to representative models of each class from C. DSA found that DD-GRU and the True system were more similar than either the NODE or the LDS model. **E)** Example fixed points from representative NODE, GRU, LDS models. Green circles represent stable FPs, red × indicate unstable FPs.

**Figure 13:**
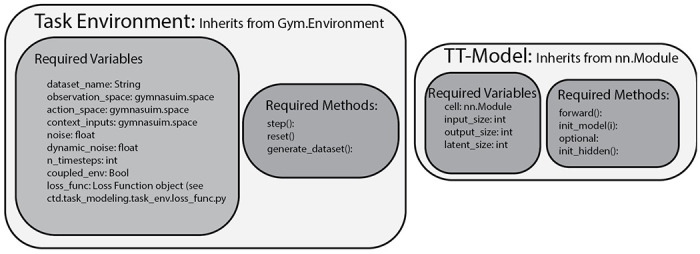
Objects for user-defined tasks and TT models

## Appendix

### A Computation-through-Dynamics Benchmark:

We envision two classes of user for CtD:

1. Data-driven models and metrics developer: Members of this group will train their data-driven models on the canonical datasets and use the metrics provided to assess the model performance. We expect this user group to be primarily interested in adding their own data-driven models and metrics to the code-base, and less interested in modifying the existing tasks or simulating their own neural data. This group, referred to as “DD-Developers” are our primary user group.
2. Task-trained model developers: Members of this group are primarily interested in adding their own tasks to the CtD Benchmark and/or simulating neural data with different characteristics (noise models, embeddings, etc.). We have provided a template class that these users can follow to incorporate new tasks into CtD, and describe the resources available to these users in Section F.1.

As described in Section 3 and shown in Figure 2A, CtD has three major components:

1. Generating canonical datasets : We provide the task environments and logic necessary to produce datasets from each of the tasks in CtD. We also include access to pre-trained models so that users can directly simulate neural data corresponding to these tasks.
2. Training data-driven models : We provide a pipeline for training neural dynamics models on simulated neural data produced by a task-trained model.
3. Comparing model performance : We provide several metrics for analyzing and comparing neural dynamics model performance, as well as the supporting code for easily logging and visualizing such results.

#### A.1 Overview of DD-Developer process flow

We provide three canonical datasets for DD-Developers. Each synthetic dataset was generated from a TT model trained to perform one of the included tasks. DD-Developers can get started with training their models on the canonical datasets by following a few simple steps:

1. Clone the github repository: Navigate to our GitHub page and clone the repository. This repository includes task-training and data-training pipelines, comparison metrics, and three pre-trained models, which are used to generate the canonical datasets.
2. Set up the environment: Follow the instructions in the README to set up the environment for the CtD Benchmark.
3. Generate the canonical datasets: Users can generate the canonical datasets using pre-trained models for each task by running the script /examples/gen_datasets.py. This script generates the canonical datasets by 1) generating inputs and outputs from a saved task-environment, 2) running the pre-trained TT model on this dataset, 3) simulating neural activity from the hidden activity of the pre-trained model (saved in /content/datasets/dd). More information on the task-environments and task-training pipeline can be found in Section B.
4. Use /examples/run_data_training.py to train models on the canonical datasets. Users can choose the model class (LFADS, SAE), the canonical dataset (NBFF, MultiTask, RandomTarget), and the input mode (supplied or inferred). Running this script without changing any parameters should start a run that will replicate the results from Figure 5. More information on data-driven models can be found in Section D.
5. Once the models are done training, users can load the saved models into Analysis_DD objects (/ctd/comparison/analysis/dd), which can be loaded into a Comparison object (/ctd/comparison/comparison.py). The Comparison object provides useful tools for visualizing and computing metrics across trained models. More information on the available comparisons can be found in Section E, and a useful example notebook can be found at /examples/compare_tt_dd_models.ipynb.

### B Canonical Datasets and Task Training:

In this section, we give details on the task environments, models and hyperparameters that were used to generate the pre-trained models for each task. We first describe the high-level Task Environment structure, and then describe how it was used for each canonical dataset. Information on using the pre-trained models to generate synthetic datasets is given in Section C. We summarize the training pipeline in Figure 7.

#### B.1 Task Environments

We use the TaskEnvironment structure to generate the task-training dataset for the task-trained model. TaskEnvironments inherit from the Gymnasium.Environment class. Each TaskEnvironment defines the logic of a given task and implements the function generate_dataset(), which returns a dictionary that contains the inputs, outputs, and other information necessary to train a model to perform the task. For TT model developers who wish to modify or add TaskEnvironments, we describe more requirements for TaskEnvironments in Section F.1.

The generate_dataset() function is the heart of the TaskEnvironment. This function returns a dictionary with the following entries:

- ics: (shape: n_samples × D). The initial conditions of the task environment (e.g., joint angles at trial start, the state of the bits in 3BFF).
- inputs: (shape: n_samples × timesteps × n_inputs_pre). The inputs to the model that can be pre-computed (e.g., inputs pulses for 3BFF).
- inputs_to_env: (shape: n_samples × timesteps × n_ext_inputs). Any inputs that are applied to the environment rather than passed directly to the TT model. The bump perturbations in the RandomTarget task are the only examples of this type of input in the provided datasets.
- targets: (shape: n_samples × timesteps × n_targets). The target values for the TT model (e.g., the true state of the 3BFF).
- true_inputs: (shape: n_samples × timesteps × n_inputs). This contains the noiseless ground truth inputs for visualization and training.
- conds: (shape: n_samples × 1). Condition indices for each trial. Used to re-weight the loss contributions of different trial types and for batching during training (used only in MultiTask).

Each TaskEnvironment also includes the loss function that is used to train models to perform the task. We describe the loss function for each of the canonical datasets in more detail below.

##### B.1.1 Three-Bit Flip-Flop (3BFF)

###### General Description

The simplest of the canonical datasets is the 3BFF dataset, which is a 3 bit memory task described first in [15]. Our implementation can be found in /task_modeling/task_env.task_env.py in the NBitFlipFlop class. Here we describe the structure of inputs and outputs of the model:

###### Environment Parameters

The NBitFlipFlop class takes the parameters described below. Values used in the canonical 3BFF dataset are given in Table 2.

- n_timesteps: The length of each trial in bins.
- noise: Standard deviation of Gaussian noise to be added to the inputs. This noise is pre-computed and the same every time a given trial is presented.
- n: The number of bits in the NBitFlipFlop (Default: 3).
- switch_prob: The probability of an input pulse on a given timestep.
- transition_blind: The number of bins after an input pulse that the loss is zeroed out (described in *Loss function* below).

**Table 2:** Parameters for the task-training and simulation datasets for 3BFF.

Task parameter	3BFF (train)	3BFF (sim)
n_trials	1000	1000
n_timesteps	500	500
noise	0.15	0.15
n	3	3
switch_prob	0.01	0.01
transition_blind	4	**NA**

###### Trial Structure

Trials begin with all states set to zero. Each timestep has a small probability of a positive or negative input pulse on each channel. Positive inputs push the state from zero to one, negative inputs push the state from one to zero, while positive (negative) inputs have no effect when the state is one (zero). We show an example trial of 3BFF in Figure 8.

###### Loss Function

During training, we penalize the mean squared error between the model output and the desired output. To avoid interference from initial transient effects, we mask the losses during the first 5 bins of each trial. Additionally, we apply a masking period (default: 4 bins, defined by transition_blind) following each input pulse. We found that this masking helped the model learn smoother dynamics that cleanly transitioned from one state to another without large discontinuous jumps in the state. This led to See /ctd/task_modeling/task_env/loss_func.py for full implementation details.

###### Task-Trained Model (NoisyGRU)

For the canonical 3BFF dataset, we used a modified Gated Recurrent Unit (GRU) network called a NoisyGRU. NoisyGRU adds a configurable intrinsic noise to the state at each time step and to the initial hidden state of the network on each trial. The noise at each time step was drawn from a standard normal distribution with mean zero and isotropic variance of 0.01, while the initial conditions were drawn from a standard normal with mean *μ* and variance of 0.05, where *μ* was a trainable parameter. This ensured that the model learned to stably encode memory regardless of semi-random initial conditions. Full hyperparameters are in Table 3. We provide the NoisyGRU model, fully-trained on 3BFF, in the /pretrained/20240503_Fig1_NBFF_NoisyGRU/ folder of the CtDB repository.

**Table 3:** NoisyGRU model parameters (3BFF)

Hyperparameters	3BFF
learning_rate	1e-3
max_epochs	500
input_size	3
output_size	3
latent_size	64
intrinsic noise	0.01
ic_noise	0.05

The full state-update equations of the NoisyGRU are:

(5)
$${\text{h}}_{0}=\mu +\eta ;\;\eta ∼𝒩\left(0,0.05\right)$$

(6)
$${\text{z}}_{t}=\sigma \left({\text{W}}_{z}{\text{x}}_{t}+{\text{U}}_{z}{\text{h}}_{t-1}+{\text{b}}_{z}\right)$$

(7)
$${\text{r}}_{t}=\sigma \left({\text{W}}_{r}{\text{x}}_{t}+{\text{U}}_{r}{\text{h}}_{t-1}+{\text{b}}_{r}\right)$$

(8)
$${\tilde{h}}_{t}=\text{tanh}\left({\text{W}}_{h}{\text{x}}_{t}+{\text{U}}_{h}\left({\text{r}}_{t}⊙h_{t-1}\right)+{\text{b}}_{h}+\xi_{t}\right);\;\xi_{t}∼𝒩\left(0,0.01\right)$$

(9)
$$h_{t}=\left(1-z_{t}\right)⊙h_{t-1}+z_{t}⊙{\tilde{h}}_{t}$$

Where ${\text{h}}_{0}$ is the initial hidden state with noise, ${\text{z}}_{t}$ is the update gate at time step t, ${\text{r}}_{t}$ is the reset gate at time step t, ${\tilde{h}}_{t}$ is the candidate hidden state at time step t, ${\text{h}}_{t}$ is the final hidden state at time step t, η and $\xi_{t}$ are the noise terms drawn from normal distributions, σ(⋅) is the sigmoid function, and ⊙ represents the element-wise (Hadamard) product.

##### B.1.2 MultiTask

###### General description

Our dataset of “intermediate” difficulty was generated using the previously described MultiTask task [33, 43], which is composed of 15 cognitive tasks that have overlapping task requirements (e.g., memory, directional responses, multiple stimulus modalities). To perform MultiTask, a model takes in 20 inputs (1 fixation input, 2 inputs for stimulus modality 1, 2 inputs for stimulus modality 2, and 15 one-hot task inputs) and has 3 outputs (1 fixation, 2 response channels). The tasks can be broken down into three categories: *response, decision making*, and *matching*.

###### Response:

During Response tasks, inputs were received on the 2 input dimensions associated with stimulus modality 1, and the model was asked to respond by producing output either in the direction of the stimulus (“Pro”) or in the opposite direction (“Anti”). Different tasks required that the model respond at different points during the trial; for “React”, the model was trained to respond immediately upon receiving the stimulus. For “Delay”, the model was trained to respond after a short delay period in which the model continues to see the stimulus. For “Memory”, the model was trained to respond after a short period when the stimulus was turned off upon receiving a go-cue. With “Pro/Anti” versions of the “React/Delay/Memory” tasks, there are 6 total tasks in the Response category.

###### Decision Making:

Decision Making tasks were designed to emulate systems that make decisions about the content of noisy inputs. In one version of the task (“IntMod”), we presented two stimuli to a single input modality, and the model was trained to move in the direction of the larger magnitude stimulus. In a second version (“ContextInt”), we presented stimuli to both modalities in each stimulation period, and models were trained to ignore one of the modalities based on the rule input. In the final version of the task, the model was trained to incorporate information from both modalities equally before deciding which was larger (“MultiModal”). With “IntMod1”, “IntMod2”, “ContextInt1”, “ContextInt2”, and “ContextIntMultimodal”, there were a total of 5 decision making tasks included in MultiTask.

###### Matching:

The final class of tasks are Matching tasks. In two of these tasks, the model must produce output that indicates whether consecutive stimuli provided to the first input modality were the same direction (“Match”) or opposite direction (“Non-Match”) of one another, responding to the second stimulus location if so. The final two Matching tasks (“MatchCat”, “MatchCatAnti”) required the model to respond only if both stimuli took on values in the range [0, π) or [π, 2π).

We show an example trial of each task in Figure 9, grouped by the task type. Lengths of each phase of the tasks are drawn from a min and max length shown in Table 4. Additional implementation details are drawn from [43]. Full task code can be found at /ctd/task_modeling/task_env/multitask.py.

**Table 4:** Task phase length by trial type: [min, max] in bins.

Trial Type	Context	Stim1	Mem1	Stim2	Response

Delay	[15, 35]	[10, 75]	[0, 0]	[0, 0]	[15, 35]
Memory	[15, 35]	[10, 80]	[10, 80]	[0, 0]	[15, 35]
React	[25, 125]	[0, 0]	[0, 0]	[0, 0]	[15, 85]
Decision	[10, 30]	[10, 80]	[10, 80]	[10, 80]	[15, 35]
Match	[10, 30]	[10, 80]	[10, 80]	[0, 0]	[15, 35]

###### Environment Parameters

The MultiTask class takes the parameters described below. The values for each parameter used when generating the canonical Multitask dataset are given in Table 5.

- n_timesteps: The maximum length across trials in bins.
- noise: Standard deviation of Gaussian noise to be added to the inputs.
- num_targets: The number of targets in the task (distributed uniformly on the circumference of the unit circle.

**Table 5:** Task-training dataset parameters (MultiTask)

Task parameter	MultiTask (train)	MultiTask (sim)
n_trials (per task)	1000	500
n_timesteps	320	320
noise	0.3	0.3
num_targets	128	32

Of note, all trials were 320 bins long (the longest possible trial given the lengths in Table 5). When feeding trials to our task-trained model, we simulated for the full 320 bins, then truncated the trials to the appropriate length for all subsequent loss computation and analysis.

###### Loss Function (MultiTask)

The MultiTask loss function was to minimize the MSE of the task-trained model outputs against the desired outputs. Because trials were of variable length, we masked the MSE loss to only include time-bins that are within each trial’s total length (i.e., the sum of the randomly drawn intervals from Table 4). We also masked the loss to exclude contributions from the first 5 bins of each trial, and the first 5 bins after the response period begins. Additionally, we re-weighted the loss contribution of the response period so that performance during this period was weighted 5 times more than the rest of the trial. This was done in accordance with [43] to help with training speed and performance.

We computed the performance of our task-trained models using the criteria described in [43], which finds the percentage of trials where the model responds in the appropriate direction (i.e. the angle was correct to within an error of < π/10 radians), or on trials without a desired response, that the model does not respond. As with the previous work, we found that our TT models consistently achieved performance that exceeded 80% success rate on the validation dataset for all tasks.

###### Task-Trained Model (NoisyGRU)

For our canonical MultiTask dataset, we used a NoisyGRU (described in B.1.1) with the hyperparameters shown in Table 6. Training batches were composed of trials from only one task. We also added a loss term that penalized the squared hidden activity that pressured activity to live near the origin, leading to simpler fixed-point structures (similar to a loss contribution used in [43]). Relevant hyperparameters for the canonical Task-trained MultiTask model are given in Table 6.

**Table 6:** NoisyGRU model parameters (MultiTask)

Hyperparameters	MultiTask
learning_rate	1e-3
max_epochs	500
input_size	20
output_size	3
latent_size	128
intrinsic noise	0.01
ic_noise	0.05
hidden_L2_penalty	1e-6

##### B.1.3 RandomTarget

###### General Description

In the RandomTarget task, a TT model is trained to control a 2 degree-of-freedom arm model actuated by 6 Hill-type muscles (analogous to pectoralis, deltoid, triceps long, triceps lat, biceps, and brachialis). By controlling the muscle activations of the arm model, the TT model must move the effector endpoint to various target locations sampled randomly from within the arm’s range of motion. TT models trained on this task receive both sensory (muscle lengths and velocities; hand endpoint position) and contextual (target position; go cue) inputs.

Each trial began with the effector endpoint at a random location in the workspace (Fig. 3E), and was composed of 3 phases 1) Context: the model held at the start location. 2) Delay: A target position (x/y) was presented to two of the context inputs of the task-trained model. 3) Reach: The target information was removed and a step-function go-cue was presented, cueing the model to reach to the target location and hold there until the end of the trial. Because information about the target location was not present after the go-cue, the TT model was obligated to remember the target location while making the reach. Additionally, we applied external bump perturbations to the hand at random times in the trial, which were randomly directed and of a variable magnitude. This resulted in reaches with and without corrective movements, thereby increasing the diversity and nature of computation required to complete the task successfully.

###### Loss Function (RandomTarget)

We trained TT models to minimize the mean squared error between the effector endpoint and the desired endpoint (i.e. the start location prior to the go-cue, and the target location after the go cue). To encourage strategies within minimal energy expenditure, the TT model was also trained to minimize and *L*_2_ norm of the muscle activations. To improve training stability, we incrementally increased the window of loss included in the training. After 200 epochs, the entire trial contributed to the training loss equally.

###### Environment Parameters

We chose parameters similar to those used in [56] for the two-link arm model. We summarize the parameters in Table 7. So that the model couldn’t anticipate the go cue and reach prior to the end of the delay period, 20% of training trials were “catch” trials in which the go-cue was never provided. During training the timing of the target presentation and go cue were random (target presentation occurred sometime in an interval of [100, 300] ms after trial start and the go cue occurred randomly between 50 ms after target presentation and the end of the trial, which lasted a total of 1.55 seconds). When simulating the neural data, the target onset and go-cue were consistent across trials (300 and 700 ms after the start of the trial, respectively), but bump timing was still random. For consistency with typical neural datasets of this type, our simulated data had consistent timings so that trials were aligned to the go-cue, rather than having occur at a random point of the trial. When we simulated the data, we trimmed the first 5 bins of each trial to ensure that the activity of the network was stable at the starting location before the reaching behavior began.

We briefly describe important parameters of the RandomTarget task here:

- n_timesteps: The length of each trial in bins.
- action_noise: Standard deviation of Gaussian noise added to the actions output by the model (i.e., motor noise).
- proprioception_noise: Standard deviation of Gaussian noise added to the proprioceptive feedback (muscle lengths and velocities)
- vision_noise: Standard deviation of Gaussian noise added to the visual feedback (endpoint position)
- context_input_noise: Standard deviation of Gaussian noise added to the context inputs (target position and go-cue).
- proprioception_delay: Simulated conduction delay of proprioceptive feedback (bins)
- vision_noise: Simulated conduction delay of visual feedback (bins)

**Table 7:** Task-training dataset parameters (RandomTarget): All timings are in bins (10 ms)

Task parameter	RandomTarget (train)	RandomTarget (sim)
n_trials	1100	1100
n_timesteps	155	155
action_noise	0.005	0.005
proprioception_noise	0.005	0.005
vision_noise	0.005	0.005
context_input_noise	0.04	0.04
proprioception_delay	2	2
vision_delay	5	5
% catch trials	20	0
% bump trials	50	50
target_on	[10,30]	30
go_cue	[targ_on +5, trial_end]	70
bump_mag	[5,10]	[5,10]

###### Task-Trained Model (NoisyGRU)

For the canonical RandomTarget dataset, we used a NoisyGRU model (described in Section B.1.1) with the hyperparameters shown in Table 8. As in Section B.1.2, we also penalized the *L*_2_ norm of the hidden activity of this model to force the activity to live closer to the origin, which produces more interesting dynamical structures.

**Table 8:** NoisyGRU model parameters (RandomTarget)

Hyperparameters	MultiTask
learning_rate	5e-3
max_epochs	2000
input_size	17
output_size	6
latent_size	128
intrinsic noise	0.01
ic_noise	0.05
hidden_L2_penalty	1e-6

### C Neural Data Simulation

Here we provide additional details on the process and implementation of the neural data simulation pipeline and provide a schematic in Figure 10.

#### C.1 Simulator

Synthetic neural data used in CtDB is simulated from hidden unit activity of a task trained model. Initially, a random subset of the task-trained model’s hidden units are sampled without replacement. The activity of each of these sampled units is standardized by subtracting the mean of each unit individually, and then normalizing by the scaled standard deviation across all units. The activity is then rectified and used to define a stochastic process from which spiking events are sampled over the course of the trial. For all of our experiments, the simulated spiking data was sampled from a Poisson process, but for flexibility we include options for sampling from distributions that are under-dispersed or over-dispersed compared to Poisson, such as the Binomial and Negative Binomial distributions, respectively. The co-smoothing bits-per-spike metric requires a set of held-out neurons in addition to a heldin set. For the canonical datasets, we sampled an additional 10 neurons from the TT network to provide these held-out neurons for computing co-bps.

The neural data simulator is called after the TT model is trained, as part of the Task Training Pipeline (more information in Sections B and F). The Simulator has parameters that control the number of artificial neurons to sample, firing rate scaling, and how hidden activity is embedded into neural rates. Parameters for the neural data simulator across tasks can be seen in Table 9.

The general API for the Simulator is as follows:

##### Variables:

- neuron_dict: dictionary containing arguments about how many neurons should be in the held-in/held-out datasets.
  - n_neurons_heldin: Number of heldin neurons to feed into DD models
  - n_neurons_heldout: Number of heldout neurons to be predicted by the DD model (in addition to heldin).
- embed_dict : dictionary containing arguments related to how the hidden unit activity of the task-trained model is processed into firing rates. Example keys include:
  - rect_func : The type of function to use for rectifying the hidden unit activity (e.g. The rate parameter of the Poisson distribution must be greater than zero.)
  - fr_scaling : Scaling factor applied to firing rates. Higher values of fr_scaling lead to lower firing rates.
- noise_dict : dictionary containing arguments related to how spiking events are sampled from the simulated firing rates. Example keys include:
  - obs_noise : *String*. For all datasets included in CtDB, we used a generalization of the Poisson distribution that considers the dispersion of the probability density function, controlled by a dispersion factor given by dispersion.
  - dispersion : Parameter controlling the level of dispersion of the probability density function. For dispersion = 1.0, the distribution is Poisson.

**Table 9:** Canonical dataset simulation parameters.

Simulation Parameters	3BFF	MultiTask	RandomTarget
n_neurons_heldin	50	50	50
n_neurons_heldout	10	10	10
rect_func	exp	exp	exp
fr_scaling	2.0	2.0	4.0
obs_noise	Poisson	Poisson	Poisson
dispersion	1.0	1.0	1.0

##### Methods:

generate_simulated_data(): Uses task-trained model to generate simulated neural responses, as well as collect additional information about the task and trials from the datamodule used to train the TT model.

##### Arguments:

- task_trained_model : Task Wrapper (described in Section F) object for the TT model after it has been fit to the task data from datamodule.
- datamodule : PyTorch Lightning Data Module for serving the task input/output data to the TT model.
- seed : Makes data generation deterministic for reproducibility (e.g. sampling which TT model hidden units to turn into neurons, sampling of spiking events, etc.)

simulate_neural_data(): Calls generate_simulated_data() and compiles all relevant fields for data-driven modeling saved as h5 files.

##### Arguments:

- task_trained_model : Task Wrapper (described in Section F) object for the TT model after it has been fit to the task data from datamodule.
- datamodule : PyTorch Lightning datamodule for serving the task input/output data to the TT model.
- run_tag : filename for saving the simulated data to the project, written in/examples/run_task_training.py
- subfolder : optional subfolder to save data somewhere more specific inside the project, specified by overrides passed to the train() function in ctd/task_modeling/task_train_prep.py
- dataset_path : highest level save path, acquired from path_dict in examples/run_task_training.py

Note that the majority of the above arguments are set at a higher level in the task training scripts and passed through to the simulator. Any direct interactions with the simulator are via configuration files.

### D Utilizing the Data-Training Pipeline

#### D.1 Training Pipeline Overview

The data training pipeline allows users to deploy models of neural dynamics on the simulated neural data produced by task-trained models. Our design allows for modular training and saving of data-driven model checkpoints and performance metrics, and supports end-to-end model training using PyTorch Lightning.

We use hydra [32] to instantiate configuration files for default model parameters, and Ray-tune [24] for hyperparameter tuning. We recommend using the template provided in ctd/data_modeling/ for adding new data-driven models or modifying existing models. See Section D.2 for details and see Figure 11 for a schematic.

A basic DD user can begin training data-driven models on the canonical datasets using the script /examples/run_data_training.py. At the top of this script, users must select from the following choices:

1. Choose the data-driven model class (SAE or LFADS).
2. Choose a model architecture (from /ctd/data_modeling/configs/{MODEL_CLASS}/{MODEL})
3. Choose a canonical dataset (options are “NBFF”, “MultiTask”, or “RandomTarget”).
4. Select whether to infer inputs or supply them to the model (INFER_INPUTS flag).

For hyperparameter sweeping, users can modify variables in SEARCH_SPACE dictionary to perform hyperparameter sweeps (e.g., tune.grid_search) on model parameters. See [24] for additional choices for hyperparameter sweeping.

#### D.2 Custom DD Model Interface

To make the addition of user-defined models more straightforward and streamlined, we provide a template class called TemplateSAE which can be found at ctd/data_modeling/models/SAE/template.py. This template inherits from the Lightning Module object in PyTorch Lightning, and has the following methods and required named outputs:

##### Required Methods:

- forward(data,inputs) : Defines the series of model operations that act on the data in an end-to-end pass through of the model. Outputs log-rates and latents.
- configure_optimizers() : Handles instantiation of optimizer. This includes determining how different subsets of parameters are trained, as defined by dictionary arguments passed to the user’s optimizer of choice. Must return a PyTorch Optimizer object.
- training_step(batch, batch_idx) : Definition of the entire end-to-end pass-through of the model, including the computation of the loss and logging of any intermediate metrics at each training step. Receives batches of data from the training dataloader defined in the datamodule. Outputs loss on training set for the current step.
- validation_step(batch, batch_idx) : Definition of how the model is evaluated on a validation set. This method often parallels training_step(), except no gradients are computed. Receives batches of data from the training dataloader defined in the datamodule. Outputs loss on validation set for the current step.

This class parallels the SAE baseline, defining all required methods and named outputs in a manner consistent with the rest of the benchmark. Users interested in defining their own models can fill out this template, including any additional custom methods as needed. Provided the user adds the corresponding config files to their repository (mentioned above in Section D.1), they should be able to directly train and analyze their models using the CtDB infrastructure!

#### D.3 Baseline Models

As part of CtDB, we provide implementations and/or comparisons for several baselines from across the literature. In the following subsections, we give brief descriptions of each approach, how hyperparameters were selected, and further motivation as to why each method was considered for CtDB.

##### D.3.1 Model Class: Generic Sequential Autoencoder (SAE)

###### Gated Recurrent Unit (GRU)

Our implementation of a GRU SAE can be found at ctd/data_modeling/models/SAE/dyn_models_GRU.py. It consists of the following components:

- **Encoder:** A bidirectional GRU processes input sequences to produce hidden states, capturing information from both past and future sequences.
- **Dropout Layers:** Applied to hidden states and initial conditions to prevent overfitting.
- **Initial Condition Linear Layer:** Maps concatenated hidden states from encoder GRU to initial condition for the decoder GRU.
- **Decoder:** An RNN with a GRU cell processes initial conditions and input sequences to produce latent states for each trial.
- **Readout Layer:** Maps latent states to output data dimensions.

The forward pass involves:

1. Passing the data through the encoder to get hidden states.
2. Applying dropout and linear mapping hidden states to the latent space.
3. Using the decoder to produce latent states from initial conditions and supplied inputs.
4. Mapping latent states to output dimensions using the readout layer.

The model uses a Poisson negative log-likelihood loss function to compute the difference between predicted log rates and target spikes, with the potential for alternative loss contributions such as weight decay, etc. The Adam optimizer is used for training, with learning rate adjustments and weight decay to regularize the model.

###### Linear Dynamical System (LDS)

We used a simplified implementation of an LDS to illustrate the utility of the metrics included in CtDB. For this, we replaced the GRU cell from Section D.3.1 with a cell that could only model simple linear dynamics, according the equations:

(10)
$$h_{t}=Ah_{t-1}+Bx_{t}+b_{h}+b_{x}$$

Where:

- $h_{t}$ is the hidden state at time step t.
- $h_{t-1}$ is the hidden state at the previous time step.
- $x_{t}$ is the input at time step t.
- A is a trainable weight matrix for the hidden state update.
- B is a trainable weight matrix for the input affecting the hidden state.
- $b_{h}$ is a trainable bias vector for the hidden state update.
- $b_{x}$ is a trainable bias vector for the input affecting the hidden state.

###### Switching LDS

The Switching Linear Dynamical System (LDS) model is designed to handle time series data exhibiting multiple regimes or states. This model dynamically switches between different linear dynamical systems to accurately capture the underlying structure of the data. Each regime is represented by its own set of parameters, allowing the model to adapt to changing patterns over time. We trained SLDS models with different numbers of constituent LDS models *K*, each representing a 128-dimensional linear dynamical system, until convergence using Expectation-Maximization to find the parameters that optimally regenerate the synthetic datasets.

For efficient training and optimization, JAX was utilized. JAX provides automatic differentiation, GPU acceleration, and vectorization of functions, significantly speeding up the training process [21]. SLDS is not currently included in CtDB, but we plan to integrate it along with other JAX-based models in future releases.

###### Neural ODE (NODE)

Our implementation of a SAE class based on Neural Ordinary Differential Equations (Neural ODEs [22]) can be found at ctd/data_modeling/models/SAE/dyn_models.py. It consists of the following components:

- **Encoder:** A bidirectional GRU processes input sequences to produce hidden states, capturing information from both past and future sequences.
- **Dropout Layers:** Applied to hidden states and initial conditions to prevent overfitting.
- **Initial Condition Linear Layer:** Maps concatenated hidden states from encoder to initial condition for the decoder.
- **Decoder:** An RNN with a Neural ODE cell processes initial conditions and input sequences to produce latent states. We describe the NODEcell in more detail below.
- **Readout Layer:** Linear mapping from latent states to output data dimensions.

The equations that define the state update for the NODE cell are:

(11)
$$h_{t+1}=h_{t}+\beta f\left(h_{t},u_{t}\right)$$

Where:

- $h_{t}$ is the hidden state at time step t.
- $u_{t}$ is the input at time step t.
- f is a function representing the change in hidden state, modeled by a Multilayer Perceptron (MLP) with a total of vf_num_layers layers that have vf_hidden_size hidden units each, and uses ReLU activations between layers.
- β is a scalar that improves training stability (set to 0.1).

Unlike typical Neural ODE models, we did not backpropagate the gradients through an ODE solver; instead, we did single-step Euler updates and used standard backpropagation-through-time to train the model. This approach has been shown to produce similar results on synthetic neural datasets as the ODE-solver-based training [55, 46]. Both forward pass and training procedure are the same as in the GRU model (Section D.3.1).

**Table 10:** Hyperparameters for data-driven models (NODE-D) from Figure 5.

Hyperparameter	NODE
encoder_size	100
latent_size	D
weight_decay	0
learning_rate	2e-3
vf_num_layers	6
vf_hidden_size	128
dropout	0.05
epochs	1000

Hyperparameters for the NODE-based SAEs shown in Figure 5C can be found in Table 10, while hyperparameters for NODE-based SAEs from Figure 12 can be found in Table 11.

##### D.3.2 Model Class: Latent Factor Analysis via Dynamical Systems (LFADS)

Latent Factor Analysis via Dynamical Systems (LFADS) [25] is a popular model that uses a latent dynamics model to reconstruct neural activity. LFADS is a sequential autencoder that uses variational training to learn the dynamics that generate a set of spiking observations. LFADS uses the same reconstruction objective as the SAE models in Section D.3.1, except LFADS also penalizes the KL divergence of the initial condition (IC) encoder against a Gaussian prior. For the CtD Benchmark we used an open source implementation of LFADS [52] for its modularity and extensibility, which we consider desirable to have compatible with CtDB and available for users as part of the benchmark. Full implementation details are given in [52].

**Table 11:** Hyperparameters for data-driven NODE models from Figure 12.

Hyperparameter	NODE
encoder_size	100
latent_size	3
weight_decay	0
learning_rate	2e-3
vf_hidden_size	128
vf_num_layers	3
dropout	0.05
epochs	1000

For variants of the SAE that are used to infer inputs, there is an additional bidirectional GRU encoder (referred to as the controller-input encoder, or CI Encoder) that outputs a sequence that is passed to another GRU referred to as the “controller”. The controller is responsible for producing time-varying inputs to the generator based on feedback from the generator at the preceding timestep, and the output of the CI encoder. The weights of the controller and CI encoder are updated to optimize the same loss as rest of the components of LFADS, while also being directly penalized by the KL divergence against a user-chosen prior distribution. In the experiments shown in Figure 6, we use a Student’s-*t* distribution (*df* = 5) to encourage the controller to infer sparse inputs. Alternative choices supported in CtDB include an Autoregressive Gaussian as well as standard Gaussian priors. Custom priors can be easily added by users. In total, the loss for training an LFADS model in CtDB is given by ℒ in Equation 12 as follows:

(12)
$$ℒ={ℒ}_{\text{Poisson}}+\lambda_{\text{con}}{ℒ}_{\text{con}}^{\text{KL}}+\lambda_{\text{IC}}{ℒ}_{\text{IC}}^{\text{KL}}$$

With $\lambda_{\mathrm{IC}}=0$ when inputs were supplied. We considered the LFADS model class as a baseline due to its customizability, enabling researchers to have an accessible but flexible model available for their initial experiments and to use for gaining intuition about Computation through Dynamics.

We provide hyperparameters for the LFADS models included in Figure 6 in Table 12. The “KL Penalty” in Section 4.3 and Figure 6 refers to the parameter kl_co_scale, where we tested how varying this parameter affected the quality of inferred dynamics.

**Table 12:** Hyperparameters for data-driven LFADS models in Figure 6.

Hyperparameter	LFADS
ic_encoder_size	128
gen_dim	128
ci_lag	1
con_dim	128
inf_inp_dim	3
fac_dim	20
cd_rate	0.3
co_prior.df	5
co_prior.loc	0
co_prior.scale	1.0
ic_prior.mean	0
ic_prior.variance	0.1
dropout	0.02
cell_clip	5.0
lr_init	1.0e-3
lr_stop	1.0e-5
lr_decay	0.95
lr_adam_beta1	0.9
lr_adam_beta2	0.999
lr_adam_episolon	1.0e-7
weight_decay	1.0e-5
kl_ic_scale	1.0e-6

### E Analysis and Comparison Methods

#### E.1 Analysis Objects

##### Task-Trained Analysis

We provide an object that abstracts the data handling away from the task-trained models or datamodules. This class Analysis_TT can be found at ctd/comparison/analysis/tt/tt.py, and contains helpful functions to obtain latent activity, compute fixed-points, and plot trials. Example usage of the Analysis_TT object can be found in examples/compare_dd_tt_models.ipynb. In addition, task-specific analysis objects can be found in the subfolder ctd/comparison/analysis/tt/tasks. These include functions to compute fixed-points during specific tasks and phases in MultiTask, among other useful features.

##### Data-Driven Analysis

We also provide an analogous object for data-driven models called Analysis_DD, located at ctd/comparison/analysis/dd/dd.py. By passing the model class (“SAE” or “LFADS”), this model standardizes the functions used to obtain hidden activity, predicted rates, and other metrics from data-driven models of any class included in CtDB. This object also allows for easy fixed-point computation and visualization.

##### Comparison

The final object included in CtDB is the Comparison object, located at ctd/comparison/comparison.py. Using load_analysis(), this object takes in Analysis objects (either task-trained or data-driven) and allows automated comparisons of metrics across models. Example usage of the Comparison object can be seen in examples/compare_tt_dd_models.ipynb.

#### E.2 Fixed Point Finding

As discussed in Section 3.4.3, fixed point structure and character can provide an informative qualitative picture of the neural dynamics, and possibly even their goal orientation. We seek to find the points in state space that minimize the magnitude of the kinetic energy of the dynamics, given by $\text{Δ}h^{2}\sim q$ in Equation 13. The dynamics near such points are sufficiently slow to enable characterization of the dynamics through linearization. The location and stability of each of these fixed points gives us insight into the organization and nature of the features and motifs of the dynamics.

To perform fixed point finding, we use an extension of an existing fixed point finding package [23]. Using ADAM to perform stochastic gradient descent, we optimize the objective given in Equation 13.

(13)
$$\arg\;\underset{z\in Z}{\min}q\left(z,u\right);q\left(z,u\right)={\left\|f\left(z,u\right)-z\right\|}_{2}$$

Where q(z,u) represents the magnitude of the state update at state z given inputs u. Only fixed points with q less than a threshold (depending on specific model) are retained for further analysis.

We characterize fixed point stability by the magnitude and sign of the eigenvalues of the Jacobian of the state transition function, evaluated at each fixed point. In a discrete dynamical system, if all eigenvalues have magnitudes within the unit circle, the fixed point is stable; otherwise, it is unstable.

#### E.3 Dynamical Similarity Analysis (DSA)

DSA is a method that combines data-driven techniques inspired from Koopman Operator Theory with Statistical Shape Analysis to identify, from data, whether or not two dynamical systems are equivalent. Two dynamical systems f and g are equivalent, or topologically conjugate, if there exists a homeomorphism ϕ that maps one system to another as follows:

(14)
$$f=\phi^{-1}\circ g\circ \phi$$

A natural similarity metric thus could be $\min_{\phi }{\left|f\left(x\right)-\phi^{-1}\circ g\left(y\right)\circ \phi \right|}_{F}$, averaged over samples from x and y recorded in a one-to-one fashion. Unfortunately, two problems exist with this direct approach. First, ϕ can be arbitrarily nonlinear and hence hard to optimize for, especially with insufficient data. Second, the two systems could be recorded independently, which may make it difficult to align trajectories across systems. DSA circumvents these two problems by mapping each system to an arbitrarily high-dimensional linear system. Conjugacies between linear systems are themselves linear maps, hence the similarity metric becomes Procrustes Analysis over Vector Fields (PAVF):

(15)
$$d\left(A_{x},A_{y}\right)=\underset{C}{\min}{\left\|A_{x}-CA_{y}C^{-1}\right\|}_{F}$$

Typically in DSA, C is optimized over the orthogonal group, O(n), rather than the more general group of invertible matrices GL(n). This is because the metric over O(n) is a proper metric, which has yet to be proven for GL(n), and O(n) is a compact group. Importantly, [51] showed that this method can be used to identify conjugacy even when ϕ was highly nonlinear. In this paper, we used the angular form of the distance, which scales from 0 (most similar) to *π*/2 (most dissimilar). In practice, however the full range is not always used, and so relative distances are typically emphasized over absolute.

According to Koopman Operator Theory [6], dynamical systems can be linearized by mapping them into a high-(oftentimes infinite-)dimensional space. Methods that seek to approximate the Koopman Operator are called the Dynamic Mode Decomposition, which is used in DSA [17]. A theorem in Koopman Operator Theory suggests that the eigenspectrum of the operators confers conjugacy [12], hence other work has used the 2-Wasserstein Distance of the operator eigenvalues to compare [58], but we found that the PAVF does a better job at differentiating.

To demonstrate how DSA differs from our linear methods, we show that DSA can ignore potentially trivial geometric distortions between the TT-and DD models, whereas the linear methods suggest that the DD model has invented features (Supp. Fig. 12).

##### E.3.1 Picking Hyperparameters

In practice, at least two main hyperparameters are required in delay-embedded DSA, which we chose to use here for its speed. First, the number of delays must be chosen to create the high dimensional embedding–we swept a large range and did not find a significant change in the results. Second, low-rank regression is used to fit the dynamics matrices, hence the rank must be chosen. Here, we chose to reduce dimensionality of each of our systems first with PCA to the top 5 components , for which previous work suggests choosing a full rank dynamics matrix. However, if intuition is lacking, predictivity metrics can be used to help select hyperparameters.

### F Utilizing the Task-Training Pipeline

We used PyTorch Lightning to modularize the code-base to allow for easy additions and extensions from users. The CtDB pipeline defines five objects designed to simplify the experience for the user. These objects are:

1. TaskEnvironment: The task environment (described in F.1, schematic in Fig. 13), contains the task logic for a given task. TaskEnvironment inherits from Gym.Environment [54], meaning that new TaskEnvironments must implement the step() and reset() functions. Additionally, it must implement the generate_dataset() function, which generates a dictionary of inputs and desired outputs for the task-trained model.
2. TT model: Inherits from nn.Module [30]. This object (schematic in Fig. 13) defines the model that will be trained to perform the task. This object must implement the forward() method, which takes in the input and the hidden state at time *t* to output the hidden state at time *t* + 1. Additionally, this object must implement the init_model() method and (optionally), the init_hidden() method.
3. TaskWrapper: Inherits from LightningModule [27]. This object takes in the TaskEnvironment and the TT model and handles the training and validation loops. This object should not need to be modified by the typical user.
4. TTDatamodule: Inherits from LightningDataModule [27]. This object handles the data generation from the TaskEnvironment and batching during training and validation. This object should not need to be modified by the typical user.
5. Simulator: This object handles the generation of synthetic neural activity from the trained TT model. The --init--() method of this object takes in two dictionaries, noise_dict and embed_dict, which provide settings for the noise model used to sample spiking events from the simulated firing rates and the nonlinear rectification/embedding of the task-trained model’s hidden unit activity, respectively. The Simulator object should not need to be modified by the typical user. We provide more detail on the Simulator in Section C.

#### F.1 Task Environment Requirements

Our task environments adhere to a general format that is modular and easily extensible, allowing researchers to add new tasks to CtDB without major code restructuring. TaskEnvironments are subclasses of Gymnasium Gym environments [54]. In total, new task environments must meet the following requirements:

##### Required Variables:

- dataset_name: *String*. Used as an identifier when saving and loading datasets in the task-training and data-training pipelines.

##### Input/Outputs:

These variables define the input/output spaces of the task. All are built using Gymnasium Space objects.

- action_space: *Gymnasium Space*. Defines the allowable range of actions to be taken in the environment. 3BFF, MultiTask, and RandomTarget have 3, 3, and 6, respectively.
- observation_space: *Gymnasium Space*. Defines the allowable range of “sensory” inputs that the environment provides. 3BFF, MultiTask, and RandomTarget have 3, 20, and 14, respectively.
- context_inputs: *Gymnasium Space*. Defines the allowable range of “context” inputs to the model. Only RandomTarget currently has non-zero context inputs (3).

##### Additional Variables:

- noise: *float*. Describes the standard deviation of Gaussian noise to be added to the inputs in the pre-generated dataset.
- n_timesteps: *int*. Provides the trial length for the TaskEnvironment (in bins).
- coupled_env: *Bool*. Indicates whether the actions need to be provided to the environment to generate the next step of sensory inputs (i.e., if the controller and environment are coupled). Only True for RandomTarget for canonical datasets.
- loss_func: A CtDB class that takes in a loss_dict and computes the loss for the environment. See /ctd/task_modeling/task_env/loss_func.py for implementations of each TaskEnvironment loss.

##### Required Methods:

- step(self, action): Inherited from Gym. Should implement the state update for a given TaskEnvironment.
- reset(self): Inherited from Gym. Should reset required variables to restart trial (e.g., set initial states, etc.).
- generate_dataset(self, n_samples): Defines how many trials should be generated from the dataset; for MultiTask, n_samples defines the number of trials to be generated for each sub-task. generate_dataset should return a dictionary the following fields given in Section B.1.

We provide a template TaskEnvironment in /ctd/task_modeling/task_env/task_env.py

### G Compute Resources

We used an internal computing cluster with a total of 30 Nvidia GeForce RTX 2080 Ti GPUs for model training. The task-training pipeline took 0.5, 8, and 4 hours to train for the 3BFF, MultiTask, and RandomTarget models, respectively. The RandomTarget task does not parallelize, due to its serial nature, so we did not use GPUs to train that model. For the data-driven models, both the SAE and LFADS models each took less than 1 hour to train; we trained a total of 95 data-driven models across all figures. With 2 models training on each GPU, all experiments took <150 GPU-hours. FP finding was fast, requiring 1 minute for each model.

### H Open-source Packages Used

- torch [30] (BSD license)
- pytorch-lightning [27] (Apache 2.0 license)
- ray.tune [24] (Apache 2.0 license)
- lfads-torch [52] (Apache 2.0 license)
- hydra [32] (MIT license)

## References

[1] Brown Thomas Graham. “The intrinsic factors in the act of progression in the mammal”. In: Proceedings of the Royal Society of London. Series B, containing papers of a biological character 84.572 (1911), pp. 308–319.

[2] Lorenz Edward N. “Deterministic nonperiodic flow”. In: Journal of atmospheric sciences 20.2 (1963), pp. 130–141.

[3] Hopfield John J. “Neural networks and physical systems with emergent collective computational abilities.” In: Proceedings of the national academy of sciences 79.8 (1982), pp. 2554–2558. 10.1073/pnas.79.8.2554 PMC346238 6953413

[4] Georgopoulos Apostolos P, Schwartz Andrew B, and Kettner Ronald E. “Neuronal population coding of movement direction”. In: Science 233.4771 (1986), pp. 1416–1419. 3749885 10.1126/science.3749885

[5] Mazor Ofer and Laurent Gilles. “Transient dynamics versus fixed points in odor representations by locust antennal lobe projection neurons”. In: Neuron 48.4 (2005), pp. 661–673. 16301181 10.1016/j.neuron.2005.09.032

[6] Mezić Igor. “Spectral properties of dynamical systems, model reduction and decompositions”. In: Nonlinear Dynamics 41 (2005), pp. 309–325.

[7] Roy Olivier and Vetterli Martin. “The effective rank: A measure of effective dimensionality”. In: 2007 15th European signal processing conference. IEEE. 2007, pp. 606–610.

[8] Sussillo David and Abbott Larry F. “Generating coherent patterns of activity from chaotic neural networks”. In: Neuron 63.4 (2009), pp. 544–557. 19709635 10.1016/j.neuron.2009.07.018 PMC2756108

[9] Marr David. Vision: A computational investigation into the human representation and processing of visual information. MIT press, 2010.

[10] Mora Thierry and Bialek William. “Are biological systems poised at criticality?” In: Journal of Statistical Physics 144 (2011), pp. 268–302.

[11] Stevenson I.H. and Kording K.P.. “How advances in neural recording affect data analysis”. In: Nature neuroscience 14.2 (2011), pp. 139–142. 21270781 10.1038/nn.2731 PMC3410539

[12] Budišić Marko, Mohr Ryan, and Mezić Igor. “Applied Koopmanism”. In: Chaos: An Interdisciplinary Journal of Nonlinear Science 22.4 (Dec. 2012), p. 047510. (Visited on 05/16/2023). 10.1063/1.4772195 23278096

[13] Laje Rodrigo and Buonomano Dean V. “Robust timing and motor patterns by taming chaos in recurrent neural networks”. In: Nature neuroscience 16.7 (2013), pp. 925–933. 23708144 10.1038/nn.3405 PMC3753043

[14] Shenoy Krishna V., Sahani Maneesh, and Churchland Mark M.. “Cortical control of arm movements: a dynamical systems perspective”. In: Annual Review of Neuroscience 36 (July 8, 2013), pp. 337–359. ISSN: 1545-4126. DOI: 10.1146/annurev-neuro-062111-150509. 23725001

[15] Sussillo David and Barak Omri. “Opening the black box: Low-dimensional dynamics in high-dimensional recurrent neural networks”. In: Neural Computation 25.3 (Mar. 2013), pp. 626–649. ISSN: 08997667. DOI: 10.1162/NECO_a_00409. URL: http://www.mitpressjournals.org/doi/10.1162/NECO_a_00409 (visited on 11/09/2018). 23272922

[16] Gao Yuanjun Linear dynamical neural population models through nonlinear embeddings. arXiv:1605.08454. type: article. arXiv, Oct. 25, 2016. DOI: 10.48550/arXiv.1605.08454. arXiv: 1605.08454[q-bio,stat]. URL: http://arxiv.org/abs/1605.08454 (visited on 05/17/2022).

[17] Brunton Steven L. “Chaos as an Intermittently Forced Linear System”. In: Nature Communications 8.1 (2017), p. 19. 10.1038/s41467-017-00030-8 PMC5449398 28559566

[18] Gallego Juan A “Neural manifolds for the control of movement”. In: Neuron 94.5 (2017), pp. 978–984. 28595054 10.1016/j.neuron.2017.05.025 PMC6122849

[19] Linderman Scott “Bayesian learning and inference in recurrent switching linear dynamical systems”. In: Artificial intelligence and statistics. PMLR. 2017, pp. 914–922.

[20] Zhao Yuan and Park Memming Il. “Variational latent gaussian process for recovering single-trial dynamics from population spike trains”. In: Neural computation 29.5 (2017), pp. 1293–1316. 28333587 10.1162/NECO_a_00953

[21] Bradbury James JAX: composable transformations of Python+NumPy programs. Version 0.3.13. 2018. URL: http://github.com/google/jax.

[22] Chen Ricky T. Q. “Neural Ordinary Differential Equations”. In: NIPs 109 (NeurIPS June 19, 2018), pp. 31–60. ISSN: 20385757. arXiv: 1806.07366. URL: https://arxiv.org/abs/1806.07366v5 (visited on 11/18/2021).

[23] Golub Matthew D. and Sussillo David. “FixedPointFinder: A Tensorflow toolbox for identifying and characterizing fixed points in recurrent neural networks”. In: Journal of Open Source Software 3.31 (Nov. 1, 2018), p. 1003. ISSN: 2475-9066. DOI: 10.21105/joss.01003. URL: https://joss.theoj.org/papers/10.21105/joss.01003 (visited on 04/04/2022).

[24] Liaw Richard Tune: A Research Platform for Distributed Model Selection and Training. July 13, 2018. arXiv: 1807.05118[cs,stat]. URL: http://arxiv.org/abs/1807.05118 (visited on 05/22/2023).

[25] Pandarinath Chethan “Inferring single-trial neural population dynamics using sequential auto-encoders”. In: Nat. Methods 15.10 (Oct. 2018), pp. 805–815. 30224673 10.1038/s41592-018-0109-9 PMC6380887

[26] Perich Matthew G, Gallego Juan A, and Miller Lee E. “A neural population mechanism for rapid learning”. In: Neuron 100.4 (2018), pp. 964–976. 30344047 10.1016/j.neuron.2018.09.030 PMC6250582

[27] William Falcon and The PyTorch Lightning team. PyTorch Lightning. Version 1.4. Mar. 2019. DOI: 10.5281/zenodo.3828935. URL: https://github.com/Lightning-AI/lightning (visited on 05/22/2023).

[28] Fujii Keisuke and Kawahara Yoshinobu. “Dynamic mode decomposition in vector-valued reproducing kernel Hilbert spaces for extracting dynamical structure among observables”. en. In: Neural Networks 117 (Sept. 2019), pp. 94–103. 31132607 10.1016/j.neunet.2019.04.020

[29] Koppe Georgia “Identifying nonlinear dynamical systems via generative recurrent neural networks with applications to fMRI”. In: PLOS Computational Biology 15.8 (Aug. 2019), pp. 1–35. DOI: 10.1371/journal.pcbi.1007263. URL: https://doi.org/10.1371/journal.pcbi.1007263. PMC6719895 31433810

[30] Paszke Adam PyTorch: An Imperative Style, High-Performance Deep Learning Library. arXiv:1912.01703. type: article. arXiv, Dec. 3, 2019. DOI: 10.48550/arXiv.1912.01703. arXiv: 1912.01703[cs,stat]. URL: http://arxiv.org/abs/1912.01703 (visited on 05/24/2022).

[31] Strogatz Steven. Nonlinear dynamics and chaos: with applications to physics, biology, chemistry, and engineering. Second edition, first issued in hardback. A Chapman & Hall book. Boca Raton London New York: CRC Press, 2019. 513 pp. ISBN: 978-0-8133-4910-7 978-0-367-09206-1.

[32] Yadan Omry. Hydra - A framework for elegantly configuring complex applications. Github. 2019. URL: https://github.com/facebookresearch/hydra.

[33] Yang Guangyu Robert “Task representations in neural networks trained to perform many cognitive tasks”. In: Nature Neuroscience 22.2 (Feb. 2019), pp. 297–306. ISSN: 1097-6256, 1546-1726. DOI: 10.1038/s41593-018-0310-2. URL: http://www.nature.com/articles/s41593-018-0310-2 (visited on 04/04/2022). 30643294 PMC11549734

[34] Vyas Saurabh “Computation Through Neural Population Dynamics”. In: Annu. Rev. Neurosci. 43 (July 2020), pp. 249–275. 32640928 10.1146/annurev-neuro-092619-094115 PMC7402639

[35] Zoltowski David, Pillow Jonathan, and Linderman Scott. “A general recurrent state space framework for modeling neural dynamics during decision-making”. In: International Conference on Machine Learning. PMLR. 2020, pp. 11680–11691.

[36] Hurwitz Cole “Targeted Neural Dynamical Modeling”. In: Advances in Neural Information Processing Systems. Ed. by Ranzato M. Vol. 34. Curran Associates, Inc., 2021, pp. 29379–29392. URL: https://proceedings.neurips.cc/paper_files/paper/2021/file/f5cfbc876972bd0d031c8abc37344c28-Paper.pdf.

[37] Jazayeri Mehrdad and Ostojic Srdjan. Interpreting neural computations by examining intrinsic and embedding dimensionality of neural activity. arXiv:2107.04084. type: article. arXiv, Aug. 27, 2021. DOI: 10.48550/arXiv.2107.04084. arXiv: 2107.04084[q-bio]. URL: http://arxiv.org/abs/2107.04084 (visited on 05/17/2022). PMC8688220 34537579

[38] Kalidindi Hari Teja “Rotational dynamics in motor cortex are consistent with a feedback controller”. In: Elife 10 (2021), e67256. 34730516 10.7554/eLife.67256 PMC8691841

[39] Kim Timothy D “Inferring latent dynamics underlying neural population activity via neural differential equations”. In: International Conference on Machine Learning. PMLR. 2021, pp. 5551–5561.

[40] Schimel Marine iLQR-VAE : control-based learning of input-driven dynamics with applications to neural data. Section: New Results Type: article. bioRxiv, Oct. 9, 2021, p. 2021.10.07.463540. DOI: 10.1101/2021.10.07.463540. URL: https://www.biorxiv.org/content/10.1101/2021.10.07.463540v1 (visited on 05/17/2022).

[41] Smith Jimmy T. H., Linderman Scott W., and Sussillo David. Reverse engineering recurrent neural networks with Jacobian switching linear dynamical systems. arXiv:2111.01256. type: article. arXiv, Nov. 1, 2021. arXiv:2111.01256[cs]. URL: http://arxiv.org/abs/2111.01256 (visited on 05/19/2022).

[42] Williams Alex H “Generalized Shape Metrics on Neural Representations”. In: Advances in Neural Information Processing Systems. Vol. 34. 2021, pp. 4738–4750. 38170102 PMC10760997

[43] Driscoll Laura, Shenoy Krishna, and Sussillo David. Flexible multitask computation in recurrent networks utilizes shared dynamical motifs. Pages: 2022.08.15.503870 Section: New Results. Aug. 15, 2022. DOI: 10.1101/2022.08.15.503870. URL: https://www.biorxiv.org/content/10.1101/2022.08.15.503870v1 (visited on 12/06/2022). PMC11239504 38982201

[44] Dubreuil Alexis “The role of population structure in computations through neural dynamics”. In: Nature neuroscience 25.6 (2022), pp. 783–794. 35668174 10.1038/s41593-022-01088-4 PMC9284159

[45] Pei Felix Neural Latents Benchmark ’21: Evaluating latent variable models of neural population activity. arXiv:2109.04463. type: article. arXiv, Jan. 17, 2022. arXiv: 2109.04463[cs,q-bio]. URL: http://arxiv.org/abs/2109.04463 (visited on 05/17/2022).

[46] Sedler Andrew R., Versteeg Christopher, and Pandarinath Chethan. “Expressive architectures enhance interpretability of dynamics-based neural population models”. In: (Dec. 7, 2022). DOI: 10.48550/arXiv.2212.03771. URL: https://arxiv.org/abs/2212.03771v1 (visited on 12/08/2022). PMC11065448 38699512

[47] Valente Adrian, Pillow Jonathan W, and Ostojic Srdjan. “Extracting computational mechanisms from neural data using low-rank RNNs”. In: Advances in Neural Information Processing Systems. Ed. by Koyejo S. Vol. 35. Curran Associates, Inc., 2022, pp. 24072–24086. URL: https://proceedings.neurips.cc/paper_files/paper/2022/file/9877d915a4b4f00e85e7b4cfdf41e450-Paper-Conference.pdf.

[48] Abbaspourazad Hamidreza “Dynamical flexible inference of nonlinear latent factors and structures in neural population activity”. In: Nature Biomedical Engineering 8 (Dec. 2023), pp. 1–24. DOI: 10.1038/s41551-023-01106-1. PMC11735406 38082181

[49] Dinc Fatih “CORNN: Convex optimization of recurrent neural networks for rapid inference of neural dynamics”. In: Thirty-seventh Conference on Neural Information Processing Systems. 2023. URL: https://openreview.net/forum?id=GGIA1p9fDT.

[50] Kim Timothy Flow-field inference from neural data using deep recurrent networks. Nov. 2023. DOI: 10.1101/2023.11.14.567136.

[51] Ostrow Mitchell “Beyond Geometry: Comparing the Temporal Structure of Computation in Neural Circuits with Dynamical Similarity Analysis”. In: Thirty-seventh Conference on Neural Information Processing Systems. 2023. URL: https://openreview.net/forum?id=7blSUMwe7R.

[52] Sedler Andrew R. and Pandarinath Chethan. lfads-torch: A modular and extensible implementation of latent factor analysis via dynamical systems. 2023. arXiv: 2309.01230 [cs.LG].

[53] Smith Jimmy T. H., Warrington Andrew, and Linderman Scott W.. Simplified State Space Layers for Sequence Modeling. 2023. arXiv: 2208.04933 [cs.LG]. URL: https://arxiv.org/abs/2208.04933.

[54] Towers Mark Gymnasium. Mar. 2023. DOI: 10.5281/zenodo.8127026. URL: https://zenodo.org/record/8127025 (visited on 07/08/2023).

[55] Versteeg Christopher “Expressive dynamics models with nonlinear injective readouts enable reliable recovery of latent features from neural activity”. In: ArXiv (2023).

[56] Codol Olivier “MotorNet: a Python toolbox for controlling differentiable biomechanical effectors with artificial neural networks”. In: eLife 12 (Mar. 7, 2024). Publisher: eLife Sciences Publications Limited. DOI: 10.7554/eLife.88591.2. URL: https://elifesciences.org/reviewed-preprints/88591 (visited on 05/28/2024). PMC11288629 39078880

[57] Qian William “Partial observation can induce mechanistic mismatches in data-constrained models of neural dynamics”. In: bioRxiv (2024), pp. 2024–05.

[58] Redman William T. Identifying Equivalent Training Dynamics. 2024. arXiv: 2302.09160 [cs.LG].

[59] Terada Yu and Toyoizumi Taro. “Chaotic neural dynamics facilitate probabilistic computations through sampling”. In: Proceedings of the National Academy of Sciences 121.18 (2024), e2312992121. 10.1073/pnas.2312992121 PMC11067032 38648479

